# Debiased machine learning for ultra-high dimensional mediation analysis

**DOI:** 10.1093/bioinformatics/btaf282

**Published:** 2025-05-05

**Authors:** Kecheng Wei, Yahang Liu, Chen Huang, Ruilang Lin, Yongfu Yu, Guoyou Qin

**Affiliations:** Department of Biostatistics, School of Public Health, Fudan University, Shanghai 200032, China; Department of Biostatistics, School of Public Health, Fudan University, Shanghai 200032, China; Department of Biostatistics, School of Public Health, Fudan University, Shanghai 200032, China; Department of Biostatistics, School of Public Health, Fudan University, Shanghai 200032, China; Department of Biostatistics, School of Public Health, Fudan University, Shanghai 200032, China; Department of Biostatistics, School of Public Health, Fudan University, Shanghai 200032, China

## Abstract

**Motivation:**

In ultra-high dimensional mediation analysis, confounding variables can influence both mediators and outcomes through complex functional forms. While machine learning (ML) approaches are effective at modeling such complex relationships, they can introduce bias when estimating mediation effects. In this article, we propose a debiased ML framework that mitigates this bias, enabling accurate identification of key mediators and precise estimation and inference of their respective contributions.

**Results:**

We construct an orthogonalized score function and use cross-fitting to reduce bias introduced by ML. To tackle ultra-high dimensional potential mediators, we implement screening and regularization techniques for variable selection and effect estimation. For statistical inference of the mediators’ contributions, we use an adjusted Sobel-type test. Simulation results demonstrate the superior performance of the proposed method in handling complex confounding. Applying this method to Alzheimer’s Disease Neuroimaging Initiative data, we identify several cytosine-phosphate-guanine sites where DNA methylation mediates the effect of body mass index on Alzheimer’s Disease.

**Availability and implementation:**

The R function DML_HDMA implementing the proposed methods is available online at https://github.com/Wei-Kecheng/DML_HDMA.

## 1 Introduction

Mediation analysis has emerged as a crucial tool for investigating the mechanisms through which exposure influences outcomes via mediators. It has been widely applied across diverse fields, including bioinformatics, economics, and social sciences, leading to important discoveries in these areas (Rijnhart *et al.* 2021).

Traditional studies have often focused on a single or a small number of mediators in the pathway from exposure to outcome (Sobel 1982). However, with the rise of “omics” technologies, such as epigenomics, high dimensional mediation analysis has gained increasing attention due to the availability of data on numerous potential mediators. For instance, Huang (2019), Liu *et al.* (2022), and Dai *et al.* (2022) proposed methods for large-scale, one-at-a-time mediation hypothesis testing. Guo *et al.* (2024), Jones *et al.* (2024), and Wang and Huang (2024) investigated dimension reduction and the estimation of total mediation effects. Song *et al.* (2020), Zhao and Luo (2022), and Wei *et al.* (2024) used regularized regression techniques to estimate the contributions of specific mediators. Furthermore, Zhang *et al.* (2016, 2021, 2024) developed frameworks for high dimensional mediation analysis that incorporate screening, regularized estimation, and inference. For a comprehensive review of these and other approaches, please refer to Zeng *et al.* (2021), Du *et al.* (2023), and Clark-Boucher *et al.* (2023).

In recent years, there has been a surge of research focused on using machine learning (ML) to estimate causal effects, particularly in the fields of causal inference and mediation analysis (Cai *et al.* 2022, Wang and Huang 2024). These innovative methodologies aim to relax traditional assumptions in causal estimation by utilizing advanced ML techniques to better capture complex data relationships. One of the most prominent approaches is the debiased (or double/orthogonal) ML framework (Chernozhukov *et al.* 2018, Kennedy 2024). Debiased ML tackles the challenge of confounder adjustment by using flexible ML techniques to estimate confounding relationships more effectively, making it particularly useful when there are numerous potential confounders or when the functional form of these effects is unknown. A key strength of debiased ML approach lies in its orthogonalized score functions and sample splitting, ensuring that the estimation of parameters of interest remains robust against biases introduced by using ML to estimate nuisance functions (Chernozhukov *et al.* 2018). Debiased ML has been applied across various disciplines, leading to further developments and extensions beyond its original scope. In mediation analysis, Farbmacher *et al.* (2022) and Xu *et al.* (2022) integrated the causal mediation framework with debiased ML, although their approach was restricted to a single mediator. Jones *et al.* (2024) later extended this framework to accommodate multiple mediators, although the number of mediators considered was still relatively small compared to the sample size, focusing primarily on estimating and inferring total mediation effects.

In this article, we adapt debiased ML for ultra-high dimensional mediation analysis, with the primary objective of identifying the key mediators from a vast set of candidate variables, estimating their mediation effects, and performing statistical inference. This approach is particularly relevant in omics studies (Zhang *et al.* 2016, 2021, 2024). For instance, in our motivating Alzheimer’s Disease Neuroimaging Initiative (ADNI) data analysis with 431 samples, we aim to examine the direct and indirect effects of body mass index (BMI) on Alzheimer’s Disease (AD), treating 865 859 DNA methylation (DNAm) cytosine-phosphate-guanine (CpG) sites as potential mediators. Our goal is to identify significant mediation sites and make inferences about their contributions. Unlike previous high dimensional mediation analysis methods, our approach integrates debiased ML techniques to improve robustness against model misspecification, facilitating valid variable selection and reliable statistical inference.

Specifically, we use structural equation modeling for mediation analysis to address two primary challenges: accounting for confounders that influence both the outcome and mediators through complex functional forms (as discussed in Section 2.1) and managing the ultra-high dimensional mediators. In Section 2.2, we introduce a debiased ML method for scenarios involving low dimensional mediators, where complex confounding functions are fitted using ML, and biases are mitigated through orthogonal score functions and cross-fitting. An adjusted Sobel-type (ASobel) method (Zhang 2025) is adopted to obtain the *P*-value and confidence interval for the mediation effect of each mediator. For ultra-high dimensional mediator scenarios, outlined in Section 2.3, we first perform a screening process to reduce dimensionality to a manageable level below the sample size, then apply regularization techniques for more precise mediator selection, and finally adopt ASobel method to conduct statistical inference for each selected mediator. Simulation results in Section 3 demonstrate that our method achieves more accurate mediation effect estimation and variable selection compared to existing approaches, under both linear and nonlinear confounding structures. In Section 4, our analysis of ADNI data identifies several CpG sites where DNAm mediates the effect of BMI on AD. Some of the genes associated with these CpG sites (e.g. NXPH1, with CpG site cg03099208) have been previously reported as differentially expressed in AD studies (Saykin *et al.* 2010). Our findings further elucidate the role of these CpG sites in mediating the relationship between BMI and AD. Section 5 concludes the research with some discussions.

## 2 Model and method

In this section, we first incorporate nonlinear functions into structural equation models to better capture complex confounding structures. We then present debiased ML estimators for the regression coefficients and their variance when the dimensionality *p* of the mediators is low. Finally, we propose a debiased ML procedure tailored for scenarios where the dimensionality *p* is ultra-high.

### 2.1 Structural equation models with complex confounding structures

Let $M_{i}={\left(M_{i}^{1},\ldots ,M_{i}^{p}\right)}^{T}$ be a p×1 vector of mediators, where $M_{i}^{j}$ is the *j*th (j=1,…,p) mediator of the *i*th (i=1,…,n) subject. Let $X_{i}\in R$, $Y_{i}\in R$, and $Z_{i}\in R^{q}$ be an exposure, an outcome, and the *q*-dimensional confounders, respectively. We consider the following structural equation models:

(1) $$Y_{i}=\alpha X_{i}+\beta^{T}M_{i}+f\left(Z_{i}\right)+e_{i},$$

 

(2) $$M_{i}=\gamma X_{i}+g\left(Z_{i}\right)+\varepsilon_{i},$$

where α, $\beta ={\left(\beta^{1},\ldots ,\beta^{p}\right)}^{T}$, and $\gamma ={\left(\gamma^{1},\ldots ,\gamma^{p}\right)}^{T}$ are the regression coefficients, f(·) and $g\left(\cdot \right)={\left(g^{1}\left(\cdot \right),\ldots ,g^{p}\left(\cdot \right)\right)}^{T}$ belong to some complicated function space. The random errors $e_{i}$ and $\varepsilon_{i}={\left(\varepsilon_{i}^{1},\ldots ,\varepsilon_{i}^{p}\right)}^{T}$ are zero-mean, where $e_{i}$ is independent of $X_{i}$, $M_{i}$, and $Z_{i}$, and $\varepsilon_{i}$ is independent of $X_{i}$ and $Z_{i}$.

In models (1) and (2), α represents the direct effect of the exposure $X_{i}$ on the outcome $Y_{i}$, while $\beta^{j}\gamma^{j}$ quantifies the indirect effect mediated through $M_{i}^{j}$. Under the assumptions of sequential ignorability and other conditions (Imai *et al.* 2010), these regression coefficients can be causally interpreted. Traditional structural equation models typically assume that f(·) and g(·) are linear functions of the confounders. We relax this assumption to account for more complex data-generating processes.

### 2.2 Debiased ML in low dimensional mediation analysis

We first consider the case where the dimension *p* of the mediators is low. Our goal is to construct high-quality point and variance estimators for ${\left(\alpha ,\beta^{T},\gamma^{T}\right)}^{T}$ when using ML approaches [such as lasso, neural networks, random forests, and ensembled version of these approaches (Hastie *et al.* 2009)] to estimate the nuisance confounding functions. However, the naive application of ML approaches directly to models (1) and (2) may result in large bias (Kennedy 2024). Motivated by the debiased ML framework of Chernozhukov *et al.* (2018) for partially linear model, for j=1,…,p and $D_{i}=\left\{X_{i},Y_{i},M_{i},Z_{i}\right\}$, we construct the following orthogonality score function:

(3) $$\begin{array}{cc} & \varphi \left(D_{i};\alpha ,\beta ,\text{f}\left(\cdot \right),\text{g}\left(\cdot \right),\text{h}\left(\cdot \right)\right)=\{Y_{i}-\text{f}\left(Z_{i}\right)\\ & -\alpha \left(X_{i}-\text{h}\left(Z_{i}\right)\right)-\beta^{T}\left(M_{i}-\text{g}\left(Z_{i}\right)\right)\}\left(\begin{array}{c} X_{i}-\text{h}\left(Z_{i}\right)\\ M_{i}-\text{g}\left(Z_{i}\right) \end{array}\right), \end{array}$$

 

(4) $$\begin{array}{cc} & \phi^{j}\left(D_{i};\gamma^{j},{\text{g}}^{j}\left(\cdot \right),\text{h}\left(\cdot \right)\right)\\ & =\left\{M_{i}^{j}-{\text{g}}^{j}\left(Z_{i}\right)-\gamma^{j}\left(X_{i}-\text{h}\left(Z_{i}\right)\right)\right\}\left(X_{i}-\text{h}\left(Z_{i}\right)\right), \end{array}$$

where $\text{f}\left(Z_{i}\right)=E\left(Y_{i}|Z_{i}\right)$, $\text{g}\left(Z_{i}\right)={\left({\text{g}}^{1}\left(Z_{i}\right),\ldots ,{\text{g}}^{p}\left(Z_{i}\right)\right)}^{T}$ with ${\text{g}}^{j}\left(Z_{i}\right)=E\left(M_{i}^{j}|Z_{i}\right)$ for j=1,…,p, and $\text{h}\left(Z_{i}\right)=E\left(X_{i}|Z_{i}\right)$ are nuisance functions.

We construct cross-fitted forms of the empirical moments to estimate ${\left(\alpha ,\beta^{T},\gamma^{T}\right)}^{T}$. Specifically, subjects {1,…,n} are randomly divided into *K* folds ${\left\{I_{k}\right\}}_{k=1}^{K}$ and the sizes for folds 1 to *K* are (⌊n/K⌋,…,⌊n/K⌋,n−(K−1)⌊n/K⌋). For each k=1,…,K, we obtain ML estimators ${\left({\hat{\text{f}}}_{k}\left(\cdot \right),{\hat{\text{g}}}_{k}{\left(\cdot \right)}^{T},{\hat{\text{h}}}_{k}\left(\cdot \right)\right)}^{T}$ based solely on the subset of data ${\left\{D_{i}\right\}}_{i\notin I_{k}}$. For j=1,…,p, the debiased ML estimator ${\left(\hat{\alpha },{\hat{\beta }}^{T},{\hat{\gamma }}^{T}\right)}^{T}$ can be obtained by solving the following empirical moments:

(5) $$\frac{1}{n}\sum_{k=1}^{K}\sum_{i\in I_{k}}\varphi \left(D_{i};\alpha ,\beta ,{\hat{\text{f}}}_{k}\left(\cdot \right),{\hat{\text{g}}}_{k}\left(\cdot \right),{\hat{\text{h}}}_{k}\left(\cdot \right)\right)=0,$$

 

(6) $$\frac{1}{n}\sum_{k=1}^{K}\sum_{i\in I_{k}}\phi^{j}\left(D_{i};\gamma^{j},{\hat{\text{g}}}_{k}^{j}\left(\cdot \right),{\hat{\text{h}}}_{k}\left(\cdot \right)\right)=0,$$

and the corresponding variance estimators for j=1,…,p is

(7) $$\begin{array}{c} {\hat{\sigma }}_{\alpha }^{2}=\text{diag}{\left(\frac{1}{n}{\hat{A}}^{-1}\hat{B}{\left({\hat{A}}^{-1}\right)}^{T}\right)}_{1},\\ {\hat{\sigma }}_{\beta^{j}}^{2}=\text{diag}{\left(\frac{1}{n}{\hat{A}}^{-1}\hat{B}{\left({\hat{A}}^{-1}\right)}^{T}\right)}_{1+j}, \end{array}$$

 

(8) $${\hat{\sigma }}_{\gamma^{j}}^{2}=\frac{1}{n}\frac{{\hat{D}}^{j}}{{\left({\hat{C}}^{j}\right)}^{2}},$$

where

$$\begin{array}{cc} \hat{A} & =-\frac{1}{n}\sum_{k=1}^{K}\sum_{i\in I_{k}}\frac{\partial \varphi \left(D_{i};\hat{\alpha },\hat{\beta },{\hat{\text{f}}}_{k}\left(\cdot \right),{\hat{\text{g}}}_{k}\left(\cdot \right),{\hat{\text{h}}}_{k}\left(\cdot \right)\right)}{\partial \left(\alpha ,\beta^{T}\right)},\\ \hat{B} & =\frac{1}{n}\sum_{k=1}^{K}\sum_{i\in I_{k}}\varphi \left(D_{i};\hat{\alpha },\hat{\beta },{\hat{\text{f}}}_{k}\left(\cdot \right),{\hat{\text{g}}}_{k}\left(\cdot \right),{\hat{\text{h}}}_{k}\left(\cdot \right)\right)\\ & \times \varphi {\left(D_{i};\hat{\alpha },\hat{\beta },{\hat{\text{f}}}_{k}\left(\cdot \right),{\hat{\text{g}}}_{k}\left(\cdot \right),{\hat{\text{h}}}_{k}\left(\cdot \right)\right)}^{T},\\ {\hat{C}}^{j} & =-\frac{1}{n}\sum_{k=1}^{K}\sum_{i\in I_{k}}\frac{\partial \phi^{j}\left(D_{i};{\hat{\gamma }}^{j},{\hat{\text{g}}}_{k}^{j}\left(\cdot \right),{\hat{\text{h}}}_{k}\left(\cdot \right)\right)}{\partial \gamma^{j}},\\ {\hat{D}}^{j} & =\frac{1}{n}\sum_{k=1}^{K}\sum_{i\in I_{k}}\phi^{j}{\left(D_{i};{\hat{\gamma }}^{j},{\hat{\text{g}}}_{k}^{j}\left(\cdot \right),{\hat{\text{h}}}_{k}\left(\cdot \right)\right)}^{2}. \end{array}$$

For the statistical inference of the regression coefficients, the 100(1−a)% Wald-type confidence intervals (CIs) and *P*-values for α, $\beta^{j}$, and $\gamma^{j}$ (j=1,…,p) are

(9) $$\begin{array}{cc} & {\text{CI}}_{\alpha }=\left[\hat{\alpha }-\Phi_{N\left(0,1\right)}^{-1}\left(1-\text{a}/2\right){\hat{\sigma }}_{\alpha },\hat{\alpha }+\Phi_{N\left(0,1\right)}^{-1}\left(1-\text{a}/2\right){\hat{\sigma }}_{\alpha }\right],\\ & {\text{CI}}_{\beta^{j}}=\left[{\hat{\beta }}^{j}-\Phi_{N\left(0,1\right)}^{-1}\left(1-\text{a}/2\right){\hat{\sigma }}_{\beta^{j}},{\hat{\beta }}^{j}+\Phi_{N\left(0,1\right)}^{-1}\left(1-\text{a}/2\right){\hat{\sigma }}_{\beta^{j}}\right],\\ & {\text{CI}}_{\gamma^{j}}=\left[{\hat{\gamma }}^{j}-\Phi_{N\left(0,1\right)}^{-1}\left(1-\text{a}/2\right){\hat{\sigma }}_{\gamma^{j}},{\hat{\gamma }}^{j}+\Phi_{N\left(0,1\right)}^{-1}\left(1-\text{a}/2\right){\hat{\sigma }}_{\gamma^{j}}\right],\\ & p_{\alpha }=2\left(1-\Phi_{N\left(0,1\right)}\left(\left|T_{\alpha }\right|\right)\right),\\ & p_{\beta^{j}}=2\left(1-\Phi_{N\left(0,1\right)}\left(\left|T_{\beta^{j}}\right|\right)\right),\\ & p_{\gamma^{j}}=2\left(1-\Phi_{N\left(0,1\right)}\left(\left|T_{\gamma^{j}}\right|\right)\right), \end{array}$$

where $\Phi_{N\left(0,1\right)}\left(\cdot \right)$ is the cumulative distribution function of the normal distribution with mean zero and standard deviation one, $\Phi_{N\left(0,1\right)}^{-1}\left(1-\text{a}/2\right)$ is the 1−a/2 quantile; $T_{\alpha }=\hat{\alpha }/{\hat{\sigma }}_{\alpha }$, $T_{\beta^{j}}={\hat{\beta }}^{j}/{\hat{\sigma }}_{\beta^{j}}$, and $T_{\gamma^{j}}={\hat{\gamma }}^{j}/{\hat{\sigma }}_{\gamma^{j}}$ are the statistics for testing α=0, $\beta^{j}=0$, and $\gamma^{j}=0$, respectively.

For the statistical inference of the mediation effect, the null and alternative hypothesis can be denoted as

(10) $$H_{0}^{j}:\beta^{j}\gamma^{j}=0\;\text{versus}\;H_{1}^{j}:\beta^{j}\gamma^{j}\neq 0.$$

The null hypothesis includes the product of parameters, which is composite and consists of the following three cases:

(11) $$\begin{array}{c} H_{0}^{j}:\{\begin{array}{c} \text{case}\;1:\beta^{j}=0,\gamma^{j}\neq 0,\\ \text{case}\;2:\beta^{j}\neq 0,\gamma^{j}=0,\\ \text{case}\;3:\beta^{j}=0,\gamma^{j}=0. \end{array} \end{array}$$

Following Zhang (2025), we calculate the *P*-value for the adjusted Sobel-type (ASobel) test, which is defined as

(12) $$\begin{array}{c} p_{\text{ASobel}}^{j}=\{\begin{array}{c} 2\left(1-\Phi_{N\left(0,1\right)}\left(\left|T_{\text{Sobel}}\right|\right)\right),\max\left\{\left|T_{\beta^{j}}\right|,\left|T_{\gamma^{j}}\right|\right\}\geq \tau ,\\ 2\left(1-\Phi_{N\left(0,0.5^{2}\right)}\left(\left|T_{\text{Sobel}}\right|\right)\right),\max\left\{\left|T_{\beta^{j}}\right|,\left|T_{\gamma^{j}}\right|\right\}<\tau , \end{array} \end{array}$$

where $T_{\text{Sobel}}={\hat{\beta }}^{j}{\hat{\gamma }}^{j}/{\hat{\sigma }}_{\beta^{j}\gamma^{j}}$ with ${\hat{\sigma }}_{\beta^{j}\gamma^{j}}=\sqrt{{\left({\hat{\beta }}^{j}{\hat{\sigma }}_{\gamma^{j}}\right)}^{2}+{\left({\hat{\gamma }}^{j}{\hat{\sigma }}_{\beta^{j}}\right)}^{2}}$ is the Sobel test statistic (Sobel 1982), and the threshold τ is chosen as $\sqrt{n}/\;\text{log}\;\left(n\right)$ following Zhang (2025). Correspondingly, the ASobel confidence interval for $\beta^{j}\gamma^{j}$ is defined as

(13) $${\text{CI}}_{\beta^{j}\gamma^{j}}=\{\begin{array}{c} {}[{\hat{\beta }}^{j}{\hat{\gamma }}^{j}-\Phi_{N\left(0,1\right)}^{-1}\left(1-\text{a}/2\right){\hat{\sigma }}_{\beta^{j}\gamma^{j}},\\ {\hat{\beta }}^{j}{\hat{\gamma }}^{j}+\Phi_{N\left(0,1\right)}^{-1}\left(1-\text{a}/2\right){\hat{\sigma }}_{\beta^{j}\gamma^{j}}],\max\left\{\left|T_{\beta^{j}}\right|,\left|T_{\gamma^{j}}\right|\right\}\geq \tau ,\\ {}[{\hat{\beta }}^{j}{\hat{\gamma }}^{j}-\Phi_{N\left(0,0.5^{2}\right)}^{-1}\left(1-\text{a}/2\right){\hat{\sigma }}_{\beta^{j}\gamma^{j}},\\ {\hat{\beta }}^{j}{\hat{\gamma }}^{j}+\Phi_{N\left(0,0.5^{2}\right)}^{-1}\left(1-\text{a}/2\right){\hat{\sigma }}_{\beta^{j}\gamma^{j}}],\max\left\{\left|T_{\beta^{j}}\right|,\left|T_{\gamma^{j}}\right|\right\}<\tau . \end{array}$$

The Bonferroni-corrected *P*-value for the ASobel test is defined as

(14) $$p_{\text{Bonf}}^{j}=\min\left(p_{\text{ASobel}}^{j}\times p,1\right).$$

If $p_{\text{Bonf}}^{j}$ is less than the significance level a, we consider $M_{i}^{j}$ to have a significant mediation effect.

Finally, if researchers wish to further investigate the characteristics of the nonparametric functions f(·) and g(·), for j=1,…,p, we define the residuals as

(15) $$\begin{array}{c} {\hat{e}}_{i}=Y_{i}-\hat{\alpha }X_{i}-{\hat{\beta }}^{T}M_{i},\\ {\hat{\varepsilon }}_{i}^{j}=M_{i}^{j}-{\hat{\gamma }}^{j}X_{i}. \end{array}$$

We treat ${\hat{e}}_{i}$ and ${\hat{\varepsilon }}_{i}^{j}$ as the responses and $Z_{i}$ as the predictors, applying a ML approach to obtain the estimated functions $\hat{f}\left(\cdot \right)$ and ${\hat{g}}^{j}\left(\cdot \right)$.

Algorithm 1 outlines the complete procedure for debiased ML in low dimensional mediation analysis. The debiased ML method has several key advantages. First, it separates the training of ML predictors for nuisance confounding functions from the calculation of debiased ML point estimation for the parameters of interest. This separation can lead to more accurate estimates, especially in ultra-high dimensional settings. In contrast, methods that simultaneously estimate confounding functions and select mediators, such as the approach in Wang and Huang (2024), could greatly increase computational complexity and instability. Second, the use of an orthogonal score function ensures that the estimation of the parameter of interest is robust to the regularization bias introduced by using ML to estimate nuisance confounding functions (Chernozhukov *et al.* 2018). Additionally, cross-fitting effectively prevents overfitting by separating the training and prediction phases for confounding functions (Chernozhukov *et al.* 2018).

Algorithm 1 Debiased ML in low dimensional mediation analysis

**Input:** Data $D_{i}=\left\{X_{i},Y_{i},M_{i},Z_{i}\right\}$ and number of folds *K*.

Ou**tput:** For j=1,…,p, the debiased ML point estimation $\hat{\alpha }$, ${\hat{\beta }}^{j}$, and ${\hat{\gamma }}^{j}$; the 100(1−α)% Wald-type confidence intervals ${\text{CI}}_{\alpha }$, ${\text{CI}}_{\beta^{j}}$, and ${\text{CI}}_{\gamma^{j}}$; the *P*-values $p_{\alpha }$, $p_{\beta^{j}}$, and $p_{\gamma^{j}}$; the *P*-value $p_{\text{ASobel}}^{j}$ for the ASobel test; the ASobel confidence interval ${\text{CI}}_{\beta^{j}\gamma^{j}}$; and the Bonferroni-corrected *P*-value $p_{\text{Bonf}}^{j}$.

1: Sample splitting. Subjects {1,…,n} are randomly divided into *K* folds ${\left\{I_{k}\right\}}_{k=1}^{K}$ and the sizes for folds 1 to *K* are (⌊n/K⌋,…,⌊n/K⌋,n−(K−1)⌊n/K⌋).

2: Train ML predictors on folds. For each k=1,…,K, using subset ${\left\{D_{i}\right\}}_{i\notin I_{k}}$ to train the ML estimators ${\left({\hat{\text{f}}}_{k}\left(\cdot \right),{\hat{\text{g}}}_{k}{\left(\cdot \right)}^{T},{\hat{\text{h}}}_{k}\left(\cdot \right)\right)}^{T}$.

3: Debiased ML. Solving empirical moments (5) and (6) to obtain the debiased ML point estimation

$$\hat{\alpha },{\hat{\beta }}^{j},\;\text{and}\;{\hat{\gamma }}^{j}\;\left(j=1,\ldots ,p\right).$$

 Calculating (7) and (8) to obtain the variance estimation ${\hat{\sigma }}_{\alpha }^{2},{\hat{\sigma }}_{\beta^{j}}^{2},\;\text{and}\;{\hat{\sigma }}_{\gamma^{j}}^{2}$ for j=1,…,p.

4: Statistical inference. For the regression coefficients, calculating (9) to obtain the 100(1−α)% Wald-type confidence intervals and *P*-values

$${\text{CI}}_{\alpha },p_{\alpha },{\text{CI}}_{\beta^{j}},p_{\beta^{j}},{\text{CI}}_{\gamma^{j}},\;\text{and}\;p_{\gamma^{j}}\;\left(j=1,\ldots ,p\right).$$

 For the mediation effect, calculating (12) to obtain the *P*-value for the ASobel test, calculating (13) to obtain the ASobel confidence interval, and calculating (14) to obtain the Bonferroni-corrected *P*-value

$$p_{\text{ASobel}}^{j},{\text{CI}}_{\beta^{j}\gamma^{j}},\;\text{and}\;p_{\text{Bonf}\;}^{j}\;\left(j=1,\ldots ,p\right).$$

### 2.3 Debiased ML in ultra-high dimensional mediation analysis

In this section, we address the scenario involving ultra-high dimensional mediators, where the number of mediators can greatly exceed the sample size. We begin by performing a screening process to reduce the dimension to a manageable level below the sample size. Following this, we apply regularization techniques to achieve more precise mediator selection. Finally, we conduct statistical inference for each selected mediator.

Step 1. (Screening). Given the ultra-high dimensional mediators, we use marginal correlation learning techniques (Fan and Lv 2008) to reduce the dimension to a moderate scale below the sample size, while ensuring that important mediators are retained with high probability. Specifically, for j=1,…,p, we consider a series of marginal models

(16) $$Y_{i}=\alpha X_{i}+\beta^{j}M_{i}^{j}+f\left(Z_{i}\right)+e_{i},$$

 

(17) $$M_{i}^{j}=\gamma^{j}X_{i}+g^{j}\left(Z_{i}\right)+\varepsilon_{i}^{j},$$

and input the data $D_{i}^{j}=\left\{X_{i},Y_{i},M_{i}^{j},Z_{i}\right\}$ into Algorithm 1 to obtain the estimated marginal effects ${\hat{\beta }}_{\text{mar}}^{j}$ and ${\hat{\gamma }}_{\text{mar}}^{j}$, where the nonparametric terms are estimated using the ML approach based on the *k*th fold dataset ${\left\{D_{i}\right\}}_{i\notin I_{k}}$ in Step 2. The index subset of mediators after screening is defined as $\tilde{J}=${*j*: $M^{j}$ is among the top ⌊n/ log (n)⌋ largest $\left|{\hat{\beta }}_{\text{mar}}^{j}{\hat{\gamma }}_{\text{mar}}^{j}\right|$ effect}, which includes important mediators with high probability.

Step 2. (Regularization). Given the moderate-dimensional mediators $M_{i}^{j}$ ($j\in \tilde{J}$), we further use regularization techniques to more accurately select mediators. Specifically, we input the data ${\tilde{D}}_{i}=\left\{X_{i},Y_{i},M_{i}^{j}(j\in \tilde{J}),Z_{i}\right\}$ into Algorithm 1, while adding an adaptive product penalty term to the empirical moment (5) as follow

(18) $$\begin{array}{cc} & \frac{1}{n}\sum_{k=1}^{K}\sum_{i\in I_{k}}\varphi \left(D_{i};\alpha ,\beta ,{\hat{\text{f}}}_{k}\left(\cdot \right),{\hat{\text{g}}}_{k}\left(\cdot \right),{\hat{\text{h}}}_{k}\left(\cdot \right)\right)\\ & +{\left\{0,\lambda {\left(\omega \;○\;\text{sgn}\left(\beta \right)\right)}^{T}\right\}}^{T}=0, \end{array}$$

where $\text{sgn}\left(\beta \right)=\left\{\text{sgn}\left(\beta^{j}\right)\right\}(j\in \tilde{J})$ is the sign function, $\omega =\left\{\omega^{j}=1/{\left|{\hat{\beta }}_{\text{ols}}^{j}{\hat{\gamma }}_{\text{ols}}^{j}\right|}^{\delta }\right\}(j\in \tilde{J})$ is an adaptive product weight for some δ>0, and ${\hat{\beta }}_{\text{ols}}^{j}$ and ${\hat{\gamma }}_{\text{ols}}^{j}$ are obtain by solving non-regularized empirical moment. The notation ω ○ sgn(β) denotes the element-wise product and λ is a regularization parameter controls the amount of shrinkage.

The adaptive product weight $\omega^{j}=1/{\left|{\hat{\beta }}_{\text{ols}}^{j}{\hat{\gamma }}_{\text{ols}}^{j}\right|}^{\delta }$ is an extension of the adaptive lasso weight (Zou 2006). The adaptive lasso weight $1/{\left|{\hat{\beta }}_{\text{ols}}^{j}\right|}^{\delta }$ only considers the contributions of $M_{i}^{j}$ in model (1), such that mediators with large $\beta^{j}$ are more likely to be selected. However, in our mediation framework, we aim to select mediators with large mediation effects, $\beta^{j}\gamma^{j}$. The adaptive product weight $1/{\left|{\hat{\beta }}_{\text{ols}}^{j}{\hat{\gamma }}_{\text{ols}}^{j}\right|}^{\delta }$ simultaneously accounts for the contributions of $M_{i}^{j}$ in models (1) and (2), making it more likely to select mediators with large $\beta^{j}\gamma^{j}$, and protecting against the removal of mediators with small $\beta^{j}$ but large $\gamma^{j}$. The regularized estimator is denoted as ${\hat{\beta }}_{\text{reg}}^{j}$, and the index subset of mediators after regularization is defined as $\tilde{\tilde{J}}=\left\{j\in \tilde{J}:{\hat{\beta }}_{\text{reg}}^{j}\neq 0\right\}$.

Step 3. (Estimation and inference). Given the relatively low dimensional mediators $M_{i}^{j}(j\in \tilde{\tilde{J}})$, we input the data ${\tilde{\tilde{D}}}_{i}=\left\{X_{i},Y_{i},M_{i}^{j}(j\in \tilde{\tilde{J}}),Z_{i}\right\}$ into Algorithm 1 to obtain the debiased ML point estimation $\hat{\alpha }$, ${\hat{\beta }}^{j}$, and ${\hat{\gamma }}^{j}$; the 100(1−a)% Wald-type confidence intervals ${\text{CI}}_{\alpha }$, ${\text{CI}}_{\beta^{j}}$, and ${\text{CI}}_{\gamma^{j}}$; the *P*-values $p_{\alpha }$, $p_{\beta^{j}}$, and $p_{\gamma^{j}}$; the *P*-value $p_{\text{ASobel}}^{j}$ for the ASobel test; the ASobel confidence interval ${\text{CI}}_{\beta^{j}\gamma^{j}}$, and the Bonferroni-corrected *P*-value $p_{\text{Bonf}}^{j}$.

Algorithm 2 outlines the complete procedure for debiased ML in ultra-high dimensional mediation analysis, which is an extension of Algorithm 1, with additional steps for screening and regularization to achieve dimension reduction.

Algorithm 2 Debiased ML in ultra-high dimensional mediation analysis

**Input:** Data $D_{i}=\left\{X_{i},Y_{i},M_{i},Z_{i}\right\}$ and number of folds *K*.

Ou**tput:** The index subset $\tilde{\tilde{J}}$ of mediators after screening and regularization. For $j\in \tilde{\tilde{J}}$, the debiased ML point estimation $\hat{\alpha }$, ${\hat{\beta }}^{j}$, and ${\hat{\gamma }}^{j}$; the 100(1−α)% Wald-type confidence intervals ${\text{CI}}_{\alpha }$, ${\text{CI}}_{\beta^{j}}$, and ${\text{CI}}_{\gamma^{j}}$; the *P*-values $p_{\alpha }$, $p_{\beta^{j}}$, and $p_{\gamma^{j}}$; the *P*-value $p_{\text{ASobel}}^{j}$ for the ASobel test; the ASobel confidence interval ${\text{CI}}_{\beta^{j}\gamma^{j}}$; and the Bonferroni-corrected *P*-value $p_{\text{Bonf}}^{j}$.

1: Screening. For j=1,…,p, considering a series of marginal models (16) and (17), and input the data $D_{i}^{j}=\left\{X_{i},Y_{i},M_{i}^{j},Z_{i}\right\}$ into Algorithm 1 to obtain the estimated marginal effects ${\hat{\beta }}_{\text{mar}}^{j}$ and ${\hat{\gamma }}_{\text{mar}}^{j}$. The index subset of mediators after screening is defined as

$$\begin{array}{cc} \tilde{J}= & \{j:M^{j}\;\text{is}\;\text{among}\;\text{the}\;\text{top}\;\left\lfloor n/\;\text{log}\;\left(n\right)\right\rfloor \\ & \text{largest}\;\left|{\hat{\beta }}_{\text{mar}}^{j}{\hat{\gamma }}_{\text{mar}}^{j}\right|\;\text{effect}\}. \end{array}$$

2: Regularization. Inputting the data ${\tilde{D}}_{i}=\left\{X_{i},Y_{i},M_{i}^{j}(j\in \tilde{J}),Z_{i}\right\}$ into Algorithm 1, while adding an adaptive product penalty term to the empirical moment as (18). The regularized estimator is denoted as ${\hat{\beta }}_{\text{reg}}^{j}$, and the index subset of mediators after regularization is defined as

$$\tilde{\tilde{J}}=\left\{j\in \tilde{J}:{\hat{\beta }}_{\text{reg}}^{j}\neq 0\right\}.$$

3: Estimation and inference. Inputting the data ${\tilde{\tilde{D}}}_{i}=\left\{X_{i},Y_{i},M_{i}^{j}(j\in \tilde{\tilde{J}}),Z_{i}\right\}$ into Algorithm 1 to obtain the debiased ML point estimation

$$\hat{\alpha },{\hat{\beta }}^{j},\;\text{and}\;{\hat{\gamma }}^{j}\;(j\in \tilde{\tilde{J}}),$$

  the 100(1−α)% Wald-type confidence intervals and *P*-values

$${\text{CI}}_{\alpha },p_{\alpha },{\text{CI}}_{\beta^{j}},p_{\beta^{j}},{\text{CI}}_{\gamma^{j}},\;\text{and}\;p_{\gamma^{j}}\;(j\in \tilde{\tilde{J}}),$$

  as in (9) for the regression coefficients, the *P*-value for the ASobel test, the ASobel confidence interval, and the Bonferroni-corrected *P*-value

$$p_{\text{ASobel}}^{j},{\text{CI}}_{\beta^{j}\gamma^{j}},\;\text{and}\;p_{\text{Bonf}\;}^{j}\;(j\in \tilde{\tilde{J}}),$$

  as in (12), (13), and (14) for the mediation effect.

## 3 Simulation studies

**Figure 1.**
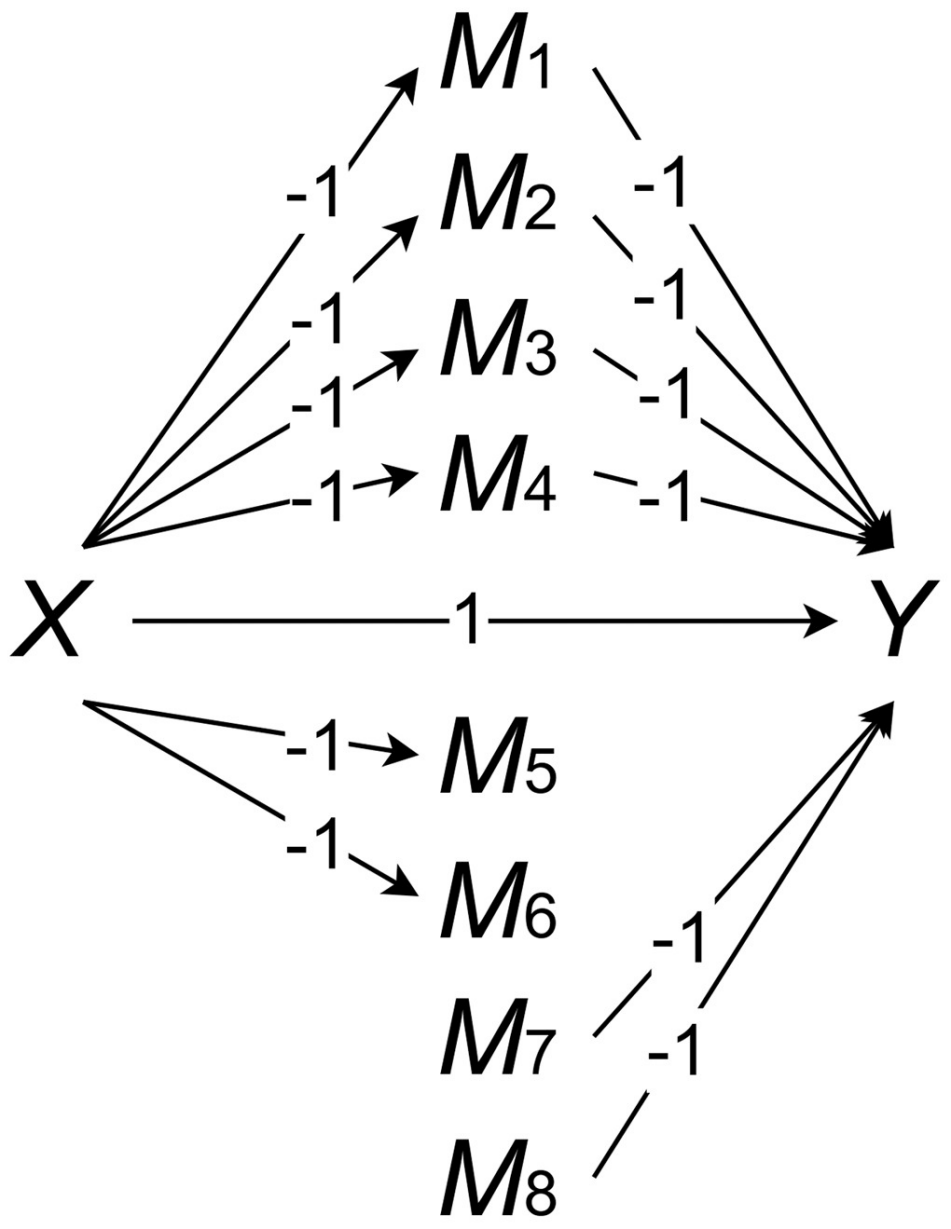
True coefficients for the simulation in structural equation models (1) and (2).

In this section, we perform comprehensive simulation studies to compare the performance of the proposed method with existing approaches. We fix q=10 and each element of the confounders $Z_{i}={\left(Z_{i}^{1},\ldots ,Z_{i}^{10}\right)}^{T}$ follows Uniform (−1,1). The random error $e_{i}∼\text{Normal}\;\left(0,0.1^{2}\right)$ and $\varepsilon_{i}^{j}∼\text{Normal}\;\left(0,0.1^{2}\right)$. The true coefficients α=1, $\beta =-{\left(1,1,1,1,0,0,1,1,\underset{p-8}{\underset{︸}{0,\ldots ,0}}\right)}^{T}$, and $\gamma =-{\left(1,1,1,1,1,1,0,0,\underset{p-8}{\underset{︸}{0,\ldots ,0}}\right)}^{T}$ are illustrated in Fig. 1.

In the simulation, we consider (i) the linear confounding structure: $f\left(Z_{i}\right)=g\left(Z_{i}\right)=h^{j}\left(Z_{i}\right)=Z_{i}^{1}+Z_{i}^{2}+Z_{i}^{3}$ for j=1,…,p, and (ii) the nonlinear confounding structure: $f\left(Z_{i}\right)=g\left(Z_{i}\right)=h^{j}\left(Z_{i}\right)=Z_{i}^{1}+{\left(Z_{i}^{2}\right)}^{2}+4I\left(Z_{i}^{3}>0\right)$ for j=1,…,p. We run Algorithm 2 to implement the proposed debiased ML (DML) method, setting the number of folds *K* to 5, as recommended by Chernozhukov *et al.* (2018). The tunings λ and δ for the adaptive lasso penalty are selected through 10-fold, 2D cross-validation. We use random forest as the ML approach, where mtry is equal to the dimension of predictors, the number of trees is set to 500. For comparison, we also implement four alternative approaches for high dimensional mediation analysis: (i) HIMA, which utilizes linear structural equation models with debiased lasso and false discovery rate control (Zhang *et al.* 2021); (ii) Bayes, which uses Bayesian linear mixed models with continuous shrinkage (Song *et al.* 2020); (iii) PLA, which uses partially linear additive models with splines and regularization (Cai *et al.* 2022); and (iv) DNN, which uses partially linear models with deep neural networks and regularization (Wang and Huang 2024).

In all scenarios, we examine sample sizes of n=200 and n=500 with dimensions p=100 and p=600, using 100 replications. Table 1 presents the estimation results for the mediation effect, including three evaluation criteria: bias, root mean square error (RMSE), and Monte Carlo standard error (MC-SE), under both linear and nonlinear confounding structures. We find that, irrespective of the confounding structure, the bias of our DML method is negligible, indicating that the proposed method effectively controls for bias introduced by various confounding structures. In contrast, the HIMA approach exhibits large bias in both linear and nonlinear settings. While the Bayes approach performs well under linear confounding structures, it shows substantial bias in nonlinear settings, suggesting that simple linear adjustments are inadequate to control for confounding bias in the presence of complex confounding structure. The PLA approach performs well under linear confounding structures but exhibits bias in nonlinear settings. A possible reason is that it approximates nonparametric functions using splines, which require a certain level of smoothness. However, the nonlinear confounding function we consider is discontinuous and does not satisfy these smoothness requirements. The DNN approach demonstrates substantial bias in both linear and nonlinear settings, as further discussed in Supplementary Section S3 of the Supplementary Data.

**Table 1.** Estimation results for the mediation effect under linear and nonlinear confounding structures.^a^

		Linear						Nonlinear					
		n=200 p=100			n=200 p=600			n=200 p=100			n=200 p=600		
		Bias	RMSE	MC-SE	Bias	RMSE	MC-SE	Bias	RMSE	MC-SE	Bias	RMSE	MC-SE
$\beta^{1}\gamma^{1}$	DML	−0.023	0.164	0.163	−0.029	0.184	0.183	−0.077	0.187	0.172	−0.056	0.190	0.182
	HIMA	0.256	0.282	0.117	0.266	0.293	0.124	−0.773	0.855	0.367	−0.795	0.885	0.392
	Bayes	−0.006	0.065	0.065	0.012	0.097	0.097	−0.964	0.967	0.067	−0.963	0.965	0.072
	PLA	−0.028	0.098	0.094	−0.090	0.157	0.130	−0.260	0.285	0.117	−0.232	0.262	0.121
	DNN	−0.629	0.629	0.031	−0.635	0.636	0.024	−0.965	0.967	0.066	−0.966	0.968	0.067
$\beta^{2}\gamma^{2}$	DML	−0.013	0.141	0.142	−0.022	0.180	0.180	−0.045	0.185	0.180	−0.052	0.181	0.174
	HIMA	0.208	0.232	0.104	0.238	0.264	0.115	−0.803	0.868	0.330	−0.818	0.891	0.355
	Bayes	−0.008	0.070	0.069	0.007	0.096	0.096	−0.963	0.965	0.067	−0.964	0.967	0.070
	PLA	−0.045	0.113	0.104	−0.096	0.159	0.127	−0.256	0.280	0.114	−0.232	0.265	0.130
	DNN	−0.628	0.629	0.029	−0.635	0.636	0.024	−0.960	0.963	0.071	−0.962	0.965	0.082
$\beta^{3}\gamma^{3}$	DML	−0.003	0.147	0.148	−0.015	0.178	0.179	−0.078	0.186	0.170	−0.048	0.187	0.182
	HIMA	0.203	0.235	0.118	0.197	0.231	0.122	−0.782	0.858	0.354	−0.806	0.878	0.350
	Bayes	0.016	0.077	0.076	−0.010	0.081	0.081	−0.959	0.962	0.072	−0.964	0.966	0.071
	PLA	−0.022	0.114	0.113	−0.096	0.154	0.120	−0.275	0.302	0.126	−0.244	0.276	0.130
	DNN	−0.629	0.630	0.031	−0.637	0.638	0.024	−0.957	0.960	0.076	−0.964	0.967	0.074
$\beta^{4}\gamma^{4}$	DML	−0.014	0.180	0.180	−0.019	0.166	0.166	−0.053	0.176	0.169	−0.081	0.195	0.178
	HIMA	0.207	0.234	0.111	0.189	0.221	0.114	−0.829	0.879	0.292	−0.863	0.908	0.286
	Bayes	0.009	0.077	0.077	−0.007	0.088	0.088	−0.963	0.965	0.071	−0.968	0.970	0.066
	PLA	−0.030	0.116	0.112	−0.119	0.186	0.144	−0.248	0.273	0.116	−0.239	0.263	0.112
	DNN	−0.628	0.629	0.032	−0.636	0.636	0.025	−0.965	0.967	0.065	−0.969	0.971	0.064
		n=500 p=100			n=500 p=600			n=500 p=100			n=500 p=600		
		Bias	RMSE	MC-SE	Bias	RMSE	MC-SE	Bias	RMSE	MC-SE	Bias	RMSE	MC-SE
$\beta^{1}\gamma^{1}$	DML	0.016	0.106	0.106	0.020	0.106	0.105	−0.030	0.100	0.096	−0.019	0.117	0.116
	HIMA	0.228	0.238	0.068	0.223	0.234	0.071	−0.403	0.405	0.039	−0.936	0.959	0.213
	Bayes	−0.006	0.041	0.040	−0.006	0.049	0.049	−0.979	0.980	0.051	−0.990	0.991	0.037
	PLA	−0.009	0.047	0.046	−0.045	0.074	0.060	−0.272	0.276	0.045	−0.260	0.268	0.064
	DNN	−0.623	0.624	0.018	−0.621	0.621	0.021	−0.975	0.977	0.063	−0.989	0.989	0.043
$\beta^{2}\gamma^{2}$	DML	0.028	0.111	0.108	0.005	0.109	0.110	−0.002	0.115	0.116	0.000	0.123	0.124
	HIMA	0.225	0.236	0.074	0.216	0.229	0.075	−0.413	0.416	0.045	−0.935	0.959	0.213
	Bayes	0.002	0.046	0.047	0.002	0.048	0.048	−0.977	0.979	0.053	−0.986	0.987	0.044
	PLA	−0.004	0.050	0.050	−0.040	0.070	0.058	−0.272	0.276	0.046	−0.264	0.271	0.059
	DNN	−0.623	0.623	0.017	−0.623	0.623	0.021	−0.970	0.973	0.075	−0.981	0.983	0.068
$\beta^{3}\gamma^{3}$	DML	0.010	0.113	0.113	0.028	0.129	0.126	−0.009	0.116	0.117	−0.019	0.107	0.106
	HIMA	0.183	0.199	0.080	0.192	0.206	0.076	−0.426	0.428	0.045	−0.913	0.951	0.265
	Bayes	0.006	0.046	0.046	−0.010	0.054	0.054	−0.976	0.977	0.054	−0.985	0.986	0.044
	PLA	0.000	0.052	0.052	−0.052	0.083	0.065	−0.265	0.269	0.052	−0.255	0.263	0.065
	DNN	−0.623	0.624	0.017	−0.621	0.621	0.023	−0.967	0.970	0.082	−0.978	0.981	0.072
$\beta^{4}\gamma^{4}$	DML	0.015	0.105	0.104	0.012	0.093	0.093	0.009	0.108	0.108	0.005	0.104	0.104
	HIMA	0.189	0.202	0.070	0.177	0.195	0.082	−0.432	0.434	0.049	−0.933	0.959	0.224
	Bayes	0.003	0.049	0.049	−0.014	0.050	0.048	−0.977	0.979	0.053	−0.986	0.987	0.044
	PLA	−0.001	0.055	0.056	−0.054	0.080	0.060	−0.274	0.280	0.055	−0.253	0.260	0.061
	DNN	−0.624	0.624	0.018	−0.622	0.622	0.021	−0.971	0.973	0.072	−0.979	0.983	0.084

a RMSE, root mean square error; MC-SE, Monte Carlo standard error. DML, debiased ML of proposed method; HIMA, linear structural equation models with debiased lasso and false discovery rate control (Zhang *et al.* 2021); Bayes, Bayesian linear mixed models with continuous shrinkage (Song *et al.* 2020); PLA, partially linear additive models with splines and regularization (Cai *et al.* 2022); DNN, partially linear models with deep neural networks and regularization (Wang and Huang 2024).

Table 2 presents the mediator selection results under both linear and nonlinear confounding structures, evaluated using two criteria: false positive rate (FPR) and false negative rate (FNR). Our DML method achieves a negligible FNR across different confounding scenarios, indicating that it successfully identifies all important mediators. The FPR is also very low, suggesting that the method rarely misidentifies unimportant mediators. In contrast, under nonlinear confounding structures, the HIMA approach frequently identifies many unimportant mediators, while the Bayes approach often fails to identify key mediators. These results highlight the limitations of simple linear adjustments in adequately controlling for confounding bias in complex settings, which significantly affects the accuracy of variable selection. The PLA approach performs well in mediator selection, however, as show in Supplementary Table S8 of the Supplementary Data, it exhibits higher FNR than our DML method when regression coefficients are small. The DNN approach often fails to identify key mediators under nonlinear confounding structures.

**Table 2.** Mediator selection results under linear and nonlinear confounding structures.^a^

	Linear				Nonlinear			
	n=200 p=100		n=200 p=600		n=200 p=100		n=200 p=600	
	FPR	FNR	FPR	FNR	FPR	FNR	FPR	FNR
DML	0.007	<0.001	0.001	0.010	0.010	<0.001	0.001	<0.001
HIMA	0.013	<0.001	0.002	<0.001	0.158	0.715	0.027	0.763
Bayes	<0.001	<0.001	<0.001	<0.001	0.039	1.000	0.006	0.998
PLA	0.001	<0.001	0.001	<0.001	0.010	0.003	0.001	<0.001
DNN	0.020	<0.001	0.003	<0.001	0.005	0.760	0.001	0.785

	n=500 p=100		n=500 p=600		n=500 p=100		n=500 p=600	
	FPR	FNR	FPR	FNR	FPR	FNR	FPR	FNR
DML	0.055	<0.001	0.006	<0.001	0.042	<0.001	0.005	<0.001
HIMA	0.020	<0.001	0.002	<0.001	0.489	<0.001	0.047	0.905
Bayes	<0.001	<0.001	<0.001	<0.001	0.027	1.000	0.005	1.000
PLA	0.001	<0.001	0.001	<0.001	0.019	<0.001	0.001	<0.001
DNN	0.021	<0.001	0.003	<0.001	0.003	0.840	<0.001	0.913

a FPR, false positive rate; FNR, false negative rate. DML, debiased ML of proposed method; HIMA, linear structural equation models with debiased lasso and false discovery rate control (Zhang *et al.* 2021); Bayes, Bayesian linear mixed models with continuous shrinkage (Song *et al.* 2020); PLA, partially linear additive models with splines and regularization (Cai *et al.* 2022); DNN, partially linear models with deep neural networks and regularization (Wang and Huang 2024).

Table 3 presents the coverage percentage of 95% confidence intervals for mediation effects under both linear and nonlinear confounding structures. For our DML method, the 95% ASobel confidence intervals for (13) achieves coverage close to the nominal 95%, with the gap narrowing as the sample size increases. This suggests that the confidence intervals constructed in (13) provide valid coverage probabilities. In contrast, other methods exhibit lower coverage percentages, particularly under nonlinear confounding structures.

**Table 3.** Coverage percentage of 95% confidence intervals for mediation effects under linear and nonlinear confounding structures.^a^

		Linear				Nonlinear			
		n=200 p=100	n=200 p=600	n=500 p=100	n=500 p=600	n=200 p=100	n=200 p=600	n=500 p=100	n=500 p=600
$\beta^{1}\gamma^{1}$	DML	0.920	0.905	0.930	0.920	0.865	0.875	0.920	0.905
	HIMA	0.060	0.080	0.010	0.030	0.020	0.030	<0.001	<0.001
	Bayes	1.000	0.980	0.990	0.970	<0.001	<0.001	<0.001	<0.001
	PLA	0.960	0.860	0.940	0.870	0.270	0.450	<0.001	0.020
	DNN	<0.001	<0.001	<0.001	<0.001	<0.001	<0.001	<0.001	<0.001
$\beta^{2}\gamma^{2}$	DML	0.940	0.915	0.885	0.915	0.890	0.890	0.910	0.890
	HIMA	0.130	0.100	0.020	0.020	0.030	<0.001	<0.001	0.010
	Bayes	0.990	0.980	0.960	0.980	<0.001	<0.001	<0.001	<0.001
	PLA	0.900	0.860	0.960	0.890	0.330	0.440	0.010	0.020
	DNN	<0.001	<0.001	<0.001	<0.001	<0.001	<0.001	<0.001	<0.001
$\beta^{3}\gamma^{3}$	DML	0.895	0.900	0.920	0.900	0.865	0.865	0.880	0.940
	HIMA	0.130	0.220	0.080	0.090	0.010	0.010	<0.001	0.010
	Bayes	0.980	0.990	0.970	0.990	<0.001	<0.001	<0.001	<0.001
	PLA	0.900	0.860	0.960	0.810	0.230	0.400	0.010	0.020
	DNN	<0.001	<0.001	<0.001	<0.001	<0.001	<0.001	<0.001	<0.001
$\beta^{4}\gamma^{4}$	DML	0.890	0.880	0.925	0.935	0.860	0.875	0.930	0.930
	HIMA	0.170	0.200	0.050	0.100	0.010	<0.001	<0.001	<0.001
	Bayes	1.000	0.990	0.970	0.980	<0.001	<0.001	<0.001	<0.001
	PLA	0.890	0.830	0.930	0.820	0.300	0.500	<0.001	0.030
	DNN	<0.001	<0.001	<0.001	<0.001	<0.001	<0.001	<0.001	<0.001

a DML, debiased ML of proposed method; HIMA, linear structural equation models with debiased lasso and false discovery rate control (Zhang *et al.* 2021); Bayes, Bayesian linear mixed models with continuous shrinkage (Song *et al.* 2020); PLA, partially linear additive models with regularization (Cai *et al.* 2022); DNN, partially linear models with deep neural networks and regularization (Wang and Huang 2024).

Additional simulation results are provided in the Supplementary Data. Specifically, Supplementary Table S1 presents the estimation and inference results for mediation effects under a simulation setting similar to that of Cai *et al.* (2022). Supplementary Table S2 reports the type I error rate and power. Supplementary Tables S3 and S4 present mediation effect estimation and mediator selection results for larger mediator dimensions (p=5000 and p=10000). Supplementary Tables S5 and S6 show results for small coefficients (α and β to be 0.1). Supplementary Tables S7 and S8 summarize results under a simulation setting closely resembling the real data structure.

## 4 ADNI data analysis

In this section, we investigate how DNAm mediates the effect of BMI on the development of AD using ADNI data. AD is a progressive neurodegenerative disorder characterized by memory loss, cognitive decline, and behavioral changes, imposing a heavy burden on older adults. BMI has been identified as an important risk factor for the development and progression of AD (Hayes *et al.* 2020, Moody *et al.* 2021), although the underlying mechanisms remain poorly understood. DNAm, a molecular modification that affects gene expression without altering the DNA sequence, can be influenced by obesity (Huang *et al.* 2018, Chen *et al.* 2021, Wang *et al.* 2023) and plays a crucial role in the progression of AD (Saykin *et al.* 2010, Reitz *et al.* 2013, Chen *et al.* 2024, Liu *et al.* 2024). Therefore, we hypothesize that DNAm may act as a pathway through which BMI influences the biological mechanisms contributing to the development of AD.

ADNI serves as a valuable data resource for the scientific community, greatly advancing our understanding of AD progression. In this analysis, which includes 431 participants, the primary exposure of interest is the baseline BMI, calculated as weight in kilograms divided by height in meters squared. The outcome of interest is the severity of AD, measured at Month 24 using the widely accepted 11-item Alzheimer’s Disease Assessment Scale (ADAS-11) cognitive score. The ADAS-11 score ranges from 0 to 70, with higher scores indicating greater severity. Due to the positive skewness observed in the score distribution, a logarithmic transformation is applied.

We include the DNAm levels of whole-genome CpG sites as candidate mediators. The DNAm profiles undergo pre-processing through the following steps: (i) excluding probes with a *P*-value <0.05, (ii) filtering out gender-related probes, (iii) removing probes with SNPs at CpG sites, (iv) eliminating cross-reactive probes, and (v) averaging DNAm levels for samples measured multiple times (Shireby *et al.* 2020). After pre-processing, 865 859 CpG sites are retained.

**Figure 2.**
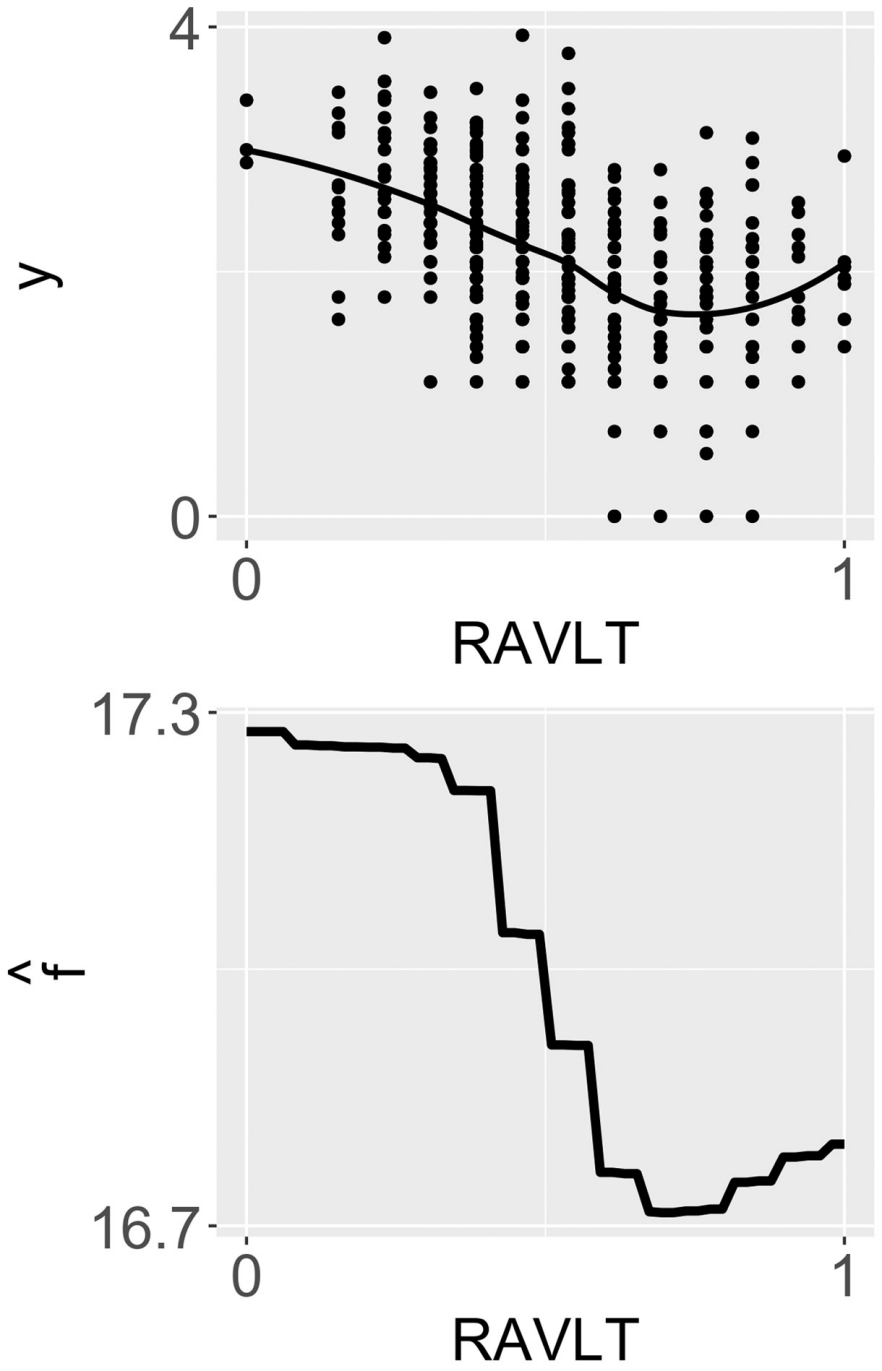
Top: relationship between the AD outcome and the RAVLT. Bottom: partial dependence plot of $\hat{f}\left(Z_{i}\right)$ with respect to RAVLT.

Following Hayes *et al.* (2020) and Moody *et al.* (2021), we consider several demographic and clinical variables measured at baseline as potential confounders, including gender (male = 1, female = 0), marital status (married = 1, other = 0), apolipoprotein ε4 carrier status (yes = 1, no = 0), age, education, Mini-Mental State Examination score, Rey Auditory-Verbal Learning Test score (RAVLT), cerebrospinal fluid tau protein level, cerebrospinal fluid Aβ42 level, and hippocampal volume. The exposure and each confounder are scaled to a 0–1 range. The top panel of Fig. 2 shows the relationship between the AD outcome and the RAVLT (plots for other confounders are presented in Supplementary Figs S1 and S2 in the Supplementary Data). The plots indicate that these relationships may be nonlinear, highlighting the importance of modeling nonlinear confounding effects.

We run Algorithm 2 to analyze the ADNI data. After the scanning step, 71 CpG sites are retained, with further regularization narrowing this down to 31 sites. Detailed information on these 31 sites is provided in Supplementary Table S9 of the Supplementary Data. Among these, 19 sites have a $p_{\text{ASobel}}$ value <0.05, and after Bonferroni correction, 9 sites remain with a $p_{\text{Bonf}}$ value <0.05, as presented in Table 4. The estimated direct effect is 0.755 (95% CI: 0.404 to 1.105), indicating that BMI has a significant direct effect on AD severity. This direct effect of BMI on AD may be due to its role in promoting inflammation, insulin resistance, cardiovascular issues, and other metabolic disturbances that negatively impact brain health (Hayes *et al.* 2020, Moody *et al.* 2021). The bottom panel of Fig. 2 shows the partial dependence plot of $\hat{f}\left(Z_{i}\right)$ with respect to RAVLT (plots for other confounders are presented in Supplementary Figs S3 and S4 in the Supplementary Data). The curves reveal that the confounders exhibit complex nonlinear effects.

**Table 4.** Summary of the selected CpG sites with $p_{\text{Bonf}}<0.05$.

CpG sites	Chromosome	Gene	${\hat{\beta }}^{j}$ (95% CI)	${\hat{\gamma }}^{j}$ (95% CI)	${\hat{\beta }}^{j}{\hat{\gamma }}^{j}$ (95% CI)	$p_{\text{ASobel}}^{j}$	$p_{\text{Bonf}}^{j}$
cg14128040	chr20	RGS19	0.620 (0.135,1.105)	0.100 (0.041,0.159)	0.062 (0.032,0.093)	<0.001	0.002
cg03099208	chr7	NXPH1	−1.216 (−2.128,−0.303)	0.048 (0.017,0.079)	−0.058 (−0.087,−0.029)	<0.001	0.003
cg13885201	chr16	ZNF23	−0.819 (−1.481,−0.158)	0.059 (0.016,0.102)	−0.048 (−0.075,−0.022)	<0.001	0.010
cg11980435	chr19	BBC3	−3.868 (−7.607,−0.130)	−0.018 (−0.028,−0.007)	0.069 (0.030,0.108)	0.001	0.017
cg27398948	chr6	SHPRH	3.934 (0.254,7.615)	−0.011 (−0.019,−0.003)	−0.045 (−0.071,−0.019)	0.001	0.025
cg17603321	chr2	RP11-214N9.1	5.096 (0.223,9.969)	−0.009 (−0.016,−0.003)	−0.048 (−0.077,−0.020)	0.001	0.026
cg27304332	chr4	SORCS2	1.267 (−0.052,2.585)	−0.038 (−0.060,−0.016)	−0.049 (−0.078,−0.020)	0.001	0.030
cg12712270	chr4	LRPAP1	−5.739 (−11.350,−0.127)	−0.008 (−0.013,−0.003)	0.044 (0.018,0.071)	0.001	0.030
cg12345696	chr5		2.481 (−0.188,5.150)	−0.020 (−0.032,−0.008)	−0.050 (−0.080,−0.019)	0.001	0.043

On the other hand, we find that BMI can indirectly influence AD severity through its effects on DNAm levels. Among the genes corresponding to the selected CpG sites, ZNF23 has been identified as one of the predictive biomarkers for white matter hyperintensities in patients with mild cognitive impairment. ZNF23 exhibits a strong correlation with immune cells and immune-related pathways, indicating its potential involvement in the pathogenesis of small-vessel injury during the early stages of AD (Chen *et al.* 2024). These findings suggest that ZNF23 may play a critical role in the immune-mediated mechanisms underlying vascular damage in AD. Our analysis indicates a negative association between ZNF23 and AD severity ($\hat{\beta }=-0.819$). NXPH1 (neurexophilin-1) is a protein involved in synaptogenesis that forms a tight complex with alpha-neurexins, a group of proteins that facilitate adhesion between dendrites and axons. This adhesion is crucial for maintaining synaptic integrity, the loss of which is a hallmark of AD (Saykin *et al.* 2010). Our findings reveal a negative association between NXPH1 and AD severity ($\hat{\beta }=-1.216$). Additionally, SORCS2 is associated with increased risk of AD, declined cognitive function and altered amyloid precursor protein processing (Reitz *et al.* 2013). Our analysis shows a positive association between SORCS2 and AD severity ($\hat{\beta }=1.267$).

For the association between BMI and DNAm, some causal relationship analyses have suggested that BMI is more likely to cause alterations in DNAm rather than being a consequence of such changes (Chen *et al.* 2021). Our results indicate that an increase in BMI may enhance methylation levels at certain sites while potentially suppressing methylation at others, as evidenced by both positive and negative $\hat{\gamma }$ in Table 4. Consequently, the estimated indirect effects for the three aforementioned CpG sites are all negative, suggesting that BMI can indirectly reduce AD severity through molecular-level DNAm. This “paradox” between direct and indirect effects has also been observed in other mediation studies on BMI and DNAm. For example, Huang *et al.* (2018) treated DNAm as a mediator of the association between mid-childhood BMI and the cardio-metabolic risk in early adolescence and observed that the mediation by mid-childhood DNAm at five CpGs was in the opposite direction to the direct effect. Similarly, Wang *et al.* (2023) examined the mediation by DNAm on the association between BMI and serum uric acid in Chinese monozygotic twins, finding that while the direct effects were all positive, the mediated effects were all negative. These opposing directions of mediation imply a complex role of DNAm in the relationship between BMI and AD severity, warranting further investigation.

## 5 Concluding remarks

In this article, we consider structural equation models with complex confounding structures. We use a debiased ML method to reduce bias from ML estimators of nuisance confounding functions. Our approach incorporates screening and regularization techniques to select relevant mediators and uses the ASobel method to evaluate the significance of the mediation effects of the selected mediators. In simulations, the proposed method demonstrates superior performance compared to existing approaches across various confounding structures. In the analysis of ADNI data, we successfully identify potential CpG sites where DNAm mediates the effect of BMI on AD.

For models (1) and (2), we use mean regression for continuous outcomes. This approach can be extended to generalized linear models for categorical outcomes (Guo *et al.* 2024), Cox models for survival data (Zhang *et al.* 2021), and quantile regression models to assess the impact of mediators across different quantiles of the outcome distribution (Zhang *et al.* 2024). Our method relaxes the linear confounding assumption of traditional structural equation models but still requires that all confounders are observed. In cases of unmeasured confounding, additional corrections, such as instrumental variables (Rudolph *et al.* 2024) or latent factor modeling (Yuan and Qu 2024), are needed. These areas will be the focus of our future research. Additional comparisons between our method and the deep learning approach of Wang and Huang (2024), as well as the debiased deep learning approach of Xu *et al.* (2022), can be found in Supplementary Sections S3 and S4 of the Supplementary Data.

In this article, we develop a debiased ML estimation and inference method for the parameters of interest, ${\left(\alpha ,\beta^{T},\gamma^{T}\right)}^{T}$, in models (1) and (2), and evaluate its performance through simulations and applications. The nonparametric functions ${\left(f\left(\cdot \right),g{\left(\cdot \right)}^{T}\right)}^{T}$ act as nuisance functions that help to more effectively adjust for confounding. If researchers wish to further explore the characteristics of the nonparametric functions, we recommend first computing the debiased ML estimator ${\left(\hat{\alpha },{\hat{\beta }}^{T},{\hat{\gamma }}^{T}\right)}^{T}$ and then applying a ML approach to the residuals in (15) to obtain ${\left(\hat{f}\left(\cdot \right),\hat{g}{\left(\cdot \right)}^{T}\right)}^{T}$. While the consistency of the estimated nonparametric functions has been established in theoretical research (e.g. the consistency of random forests in Chi *et al.* 2022), great challenges remain for conducting statistical inference on these estimates. In particular, theoretical results on the asymptotic distributions of ${\left(\hat{f}\left(\cdot \right),\hat{g}{\left(\cdot \right)}^{T}\right)}^{T}$ are still lacking, and the calculation of coverage probabilities for multidimensional nonparametric functions is complicated by the curse of dimensionality (Wager and Athey 2018). Investigating the inference properties and coverage probabilities of nonparametric functions remains an important avenue for future research.

## Supplementary Material

btaf282_Supplementary_Data

## Data Availability

Publicly available datasets were analyzed in this study. These data can be found at: adni.loni.usc.edu. The R code is publicly available on Figshare under DOI 10.6084/m9.figshare.28681211.
