# Supplementary material for: Debiased machine learning for ultra-high dimensional mediation analysis
Source: Bioinformatics. 2025 May 5;41(6):btaf282. doi: 10.1093/bioinformatics/btaf282 (PMC12198499; doi:10.1093/bioinformatics/btaf282)
Supplement: btaf282_Supplementary_Data [file btaf282_supplementary_data.pdf]

Supplementary materials for

Debiased machine learning for ultra-high dimensional mediation analysis

Kecheng Wei, Yahang Liu, Chen Huang, Ruilang Lin, Yongfu Yu and Guoyou Qin

Department of Biostatistics, School of Public Health, Fudan University,

No.130 Dong An Road, 200032, Shanghai, China

S1 Additional simulation results

We have added a simulation study similar to that of Cai et al. (2022). Specifically, we consider 10 confounders, $\boldsymbol{Z}_i = (Z_i^1, ..., Z_i^{10})^T$, each following a Uniform $(-1, 1)$ distribution. The nonlinear confounding structure is defined as $f(\boldsymbol{Z}_i) = g(\boldsymbol{Z}_i) = h^j(\boldsymbol{Z}_i) = Z_i^1 + (Z_i^2)^2 + \sin(Z_i^3)$ for $j = 1, ..., p$, where the linear effect of Z_i^1, the quadratic effect of Z_i^2, and the trigonometric effect of Z_i^3 are consistent with those in Cai et al. (2022). However, our nonlinear confounding function is more complex than that of Cai et al. (2022), as their study only incorporated two confounders, whereas we consider ten.

Table S1 presents the estimation results for the mediation effect with $n = 200$ and $p = 100$. The results indicate that our proposed DML method outperforms other approaches and achieves nominal coverage probability. Notably, since the confounding function we specify is additive and continuous, it satisfies the assumption of the PLA approach proposed by Cai et al. (2022), which also performs well.

Table S1: Estimation and inference results for mediation effects under a simulation setting similar to that of Cai et al. (2022) with $n = 200$ and $p = 100$.

		Bias	RMSE	MC-SE	CP
$\beta^1\gamma^1$	DML	-0.034	0.184	0.181	0.905
	HIMA	0.159	0.195	0.113	0.240
	Bayes	-0.142	0.191	0.128	0.605
	PLA	-0.018	0.107	0.106	0.935
	DNN	-0.574	0.574	0.028	<0.001
$\beta^2\gamma^2$	DML	-0.012	0.173	0.173	0.915
	HIMA	0.135	0.172	0.107	0.310
	Bayes	-0.146	0.194	0.128	0.640
	PLA	-0.034	0.117	0.112	0.880
	DNN	-0.576	0.576	0.027	<0.001
$\beta^3\gamma^3$	DML	0.006	0.157	0.158	0.935
	HIMA	0.124	0.166	0.111	0.340
	Bayes	-0.134	0.187	0.130	0.640
	PLA	-0.018	0.095	0.093	0.950
	DNN	-0.574	0.575	0.029	<0.001
$\beta^4\gamma^4$	DML	0.002	0.174	0.175	0.935
	HIMA	0.097	0.152	0.117	0.360
	Bayes	-0.140	0.193	0.134	0.635
	PLA	-0.026	0.109	0.106	0.925
	DNN	-0.574	0.575	0.027	<0.001

Note: RMSE, root mean square error; MC-SE, Monte Carlo standard error; CP: coverage percentage of 95% confidence intervals. DML, debiased ML of proposed method; HIMA, linear structural equation models with debiased lasso and false discovery rate control (Zhang et al., 2021); Bayes, Bayesian linear mixed models with continuous shrinkage (Song et al., 2020); PLA, partially linear additive models with splines and regularization (Cai et al., 2022); DNN, partially linear models with deep neural networks and regularization (Wang and Huang, 2024).

Table S2 reports the type I error rate and power under both linear and nonlinear confounding structures. The proposed DML method achieves high empirical power while effectively controlling the type I error rate. In contrast, other methods demonstrate lower empirical power, particularly in the presence of nonlinear confounding structures.

Table S2: Mediator selection results under linear and nonlinear confounding structures.

	linear				nonlinear			
	$n = 200$ $p = 100$		$n = 200$ $p = 600$		$n = 200$ $p = 100$		$n = 200$ $p = 600$	
	Type I	Power	Type I	Power	Type I	Power	Type I	Power
DML	0.007	1.000	0.001	0.990	0.010	1.000	0.001	1.000
HIMA	0.013	1.000	0.002	1.000	0.158	0.285	0.027	0.238
Bayes	<0.001	1.000	<0.001	1.000	0.039	<0.001	0.006	0.003
PLA	0.001	1.000	0.001	1.000	0.010	0.998	0.001	1.000
DNN	0.020	1.000	0.003	1.000	0.005	0.240	0.001	0.215
	$n = 500$ $p = 100$		$n = 500$ $p = 600$		$n = 500$ $p = 100$		$n = 500$ $p = 600$	
	Type I	Power	Type I	Power	Type I	Power	Type I	Power
DML	0.055	1.000	0.006	1.000	0.042	1.000	0.005	1.000
HIMA	0.020	1.000	0.002	1.000	0.489	1.000	0.047	0.095
Bayes	<0.001	1.000	<0.001	1.000	0.027	<0.001	0.005	<0.001
PLA	0.001	1.000	0.001	1.000	0.019	1.000	0.001	1.000
DNN	0.021	1.000	0.003	1.000	0.003	0.160	<0.001	0.088

Note: Type I, type I error rate. DML, debiased ML of proposed method; HIMA, linear structural equation models with debiased lasso and false discovery rate control (Zhang et al., 2021); Bayes, Bayesian linear mixed models with continuous shrinkage (Song et al., 2020); PLA, partially linear additive models with splines and regularization (Cai et al., 2022); DNN, partially linear models with deep neural networks and regularization (Wang and Huang, 2024).

We have included simulations with larger mediator dimensions, specifically for $p = 5000$ and $p = 10000$. Table S3 presents the estimation results for the mediation effect under both linear and nonlinear confounding structures with $n = 200$, while Table S4 presents the mediator selection results. The results indicate that our proposed DML method outperforms other approaches, including PLA and DNN, demonstrating that our method performs effectively with high-dimensional mediators.

Table S3: Estimation results for the mediation effect under linear and nonlinear confounding structures with $n = 200$ and $p = 5000$ or 10000.

		linear						nonlinear					
		$p = 5000$			$p = 10000$			$p = 5000$			$p = 10000$		
		Bias	RMSE	MC-SE	Bias	RMSE	MC-SE	Bias	RMSE	MC-SE	Bias	RMSE	MC-SE
$\beta^1\gamma^1$	DML	-0.045	0.177	0.173	-0.039	0.170	0.168	-0.045	0.140	0.134	-0.050	0.176	0.171
	HIMA	0.249	0.273	0.113	0.236	0.270	0.132	-0.904	0.942	0.269	-0.876	0.916	0.268
	Bayes	-0.008	0.099	0.100	-0.037	0.130	0.126	-0.978	0.980	0.060	-0.971	0.973	0.064
	PLA	-0.250	0.278	0.123	-0.281	0.325	0.164	-0.258	0.289	0.131	-0.255	0.283	0.124
	DNN	-0.632	0.633	0.026	-0.638	0.639	0.027	-0.979	0.981	0.061	-0.974	0.975	0.057
$\beta^2\gamma^2$	DML	-0.017	0.168	0.169	-0.001	0.161	0.163	-0.111	0.209	0.179	0.004	0.174	0.175
	HIMA	0.216	0.248	0.123	0.262	0.286	0.116	-0.908	0.944	0.262	-0.872	0.914	0.278
	Bayes	-0.027	0.129	0.127	-0.017	0.118	0.118	-0.983	0.985	0.051	-0.969	0.971	0.067
	PLA	-0.246	0.298	0.170	-0.229	0.274	0.152	-0.239	0.269	0.125	-0.206	0.231	0.107
	DNN	-0.635	0.636	0.025	-0.639	0.639	0.026	-0.983	0.984	0.055	-0.973	0.975	0.058
$\beta^3\gamma^3$	DML	0.004	0.185	0.186	-0.020	0.182	0.183	-0.047	0.190	0.186	-0.066	0.204	0.195
	HIMA	0.208	0.240	0.120	0.202	0.230	0.110	-0.906	0.943	0.262	-0.887	0.920	0.244
	Bayes	-0.003	0.109	0.110	-0.024	0.113	0.112	-0.980	0.981	0.057	-0.971	0.973	0.063
	PLA	-0.222	0.261	0.137	-0.237	0.276	0.144	-0.234	0.273	0.141	-0.244	0.272	0.123
	DNN	-0.633	0.634	0.026	-0.639	0.640	0.028	-0.977	0.979	0.070	-0.974	0.976	0.057
$\beta^4\gamma^4$	DML	-0.004	0.196	0.198	-0.012	0.162	0.163	-0.019	0.154	0.155	-0.101	0.199	0.174
	HIMA	0.187	0.219	0.116	0.199	0.226	0.108	-0.949	0.965	0.175	-0.888	0.920	0.244
	Bayes	-0.008	0.098	0.098	-0.031	0.122	0.119	-0.986	0.987	0.049	-0.971	0.973	0.062
	PLA	-0.240	0.280	0.146	-0.278	0.312	0.143	-0.242	0.264	0.106	-0.259	0.288	0.127
	DNN	-0.635	0.635	0.026	-0.638	0.639	0.027	-0.988	0.989	0.041	-0.974	0.975	0.057

Note: RMSE, root mean square error; MC-SE, Monte Carlo standard error. DML, debiased ML of proposed method; HIMA, linear structural equation models with debiased lasso and false discovery rate control (Zhang et al., 2021); Bayes, Bayesian linear mixed models with continuous shrinkage (Song et al., 2020); PLA, partially linear additive models with splines and regularization (Cai et al., 2022); DNN, partially linear models with deep neural networks and regularization (Wang and Huang, 2024).

Table S4: Mediator selection results under linear and nonlinear confounding structures with $n = 200$ and $p = 5000$ or 10000.

	linear				nonlinear			
	$p = 5000$		$p = 10000$		$p = 5000$		$p = 10000$	
	FPR	FNR	FPR	FNR	FPR	FNR	FPR	FNR
DML	<0.001	<0.001	<0.001	<0.001	<0.001	<0.001	<0.001	<0.001
HIMA	<0.001	<0.001	<0.001	<0.001	0.004	0.890	0.002	0.820
Bayes	<0.001	<0.001	<0.001	<0.001	0.001	1.000	<0.001	1.000
PLA	<0.001	0.010	<0.001	0.025	<0.001	<0.001	<0.001	<0.001
DNN	<0.001	<0.001	<0.001	<0.001	<0.001	0.895	<0.001	0.820

Note: FPR, false positive rate; FNR, false negative rate. DML, debiased ML of proposed method; HIMA, linear structural equation models with debiased lasso and false discovery rate control (Zhang et al., 2021); Bayes, Bayesian linear mixed models with continuous shrinkage (Song et al., 2020); PLA, partially linear additive models with splines and regularization (Cai et al., 2022); DNN, partially linear models with deep neural networks and regularization (Wang and Huang, 2024).

We have multiplied the outcome model $Y_i = \alpha X_i + \boldsymbol{\beta}^T \boldsymbol{M}_i + f(\boldsymbol{Z}_i) + e_i$ by 0.1, which resulted in the values α and $\boldsymbol{\beta}$ changing from their original absolute value of 1 to 0.1. Table S5 presents the estimation results for the mediation effect under both linear and nonlinear confounding structures with $n = 200$ and $p = 100$, while Table S6 presents the mediator selection results. The results indicate that our proposed DML method outperforms other approaches, demonstrating that our method performs effectively with small coefficients.

Table S5: Estimation results for the mediation effect with small coefficients under linear and nonlinear confounding structures with $n = 200$ and $p = 100$.

		linear			nonlinear		
		Bias	RMSE	MC-SE	Bias	RMSE	MC-SE
$\beta^1\gamma^1$	DML	0.002	0.018	0.018	-0.009	0.018	0.016
	HIMA	0.024	0.027	0.013	-0.082	0.088	0.034
	Bayes	-0.098	0.098	0.001	-0.098	0.098	0.006
	PLA	-0.004	0.008	0.007	-0.025	0.027	0.010
	DNN	-0.074	0.075	0.014	-0.096	0.097	0.009
$\beta^2\gamma^2$	DML	-0.002	0.016	0.016	-0.005	0.023	0.022
	HIMA	0.022	0.025	0.013	-0.084	0.089	0.030
	Bayes	-0.098	0.098	0.001	-0.099	0.099	0.003
	PLA	-0.005	0.011	0.010	-0.026	0.028	0.012
	DNN	-0.074	0.075	0.014	-0.098	0.098	0.005
$\beta^3\gamma^3$	DML	-0.002	0.019	0.019	-0.011	0.019	0.016
	HIMA	0.022	0.025	0.012	-0.079	0.088	0.039
	Bayes	-0.098	0.098	0.001	-0.098	0.098	0.007
	PLA	-0.006	0.011	0.010	-0.025	0.028	0.013
	DNN	-0.074	0.075	0.014	-0.096	0.096	0.011
$\beta^4\gamma^4$	DML	-0.001	0.015	0.016	-0.001	0.016	0.016
	HIMA	0.020	0.024	0.014	-0.086	0.091	0.030
	Bayes	-0.098	0.098	0.001	-0.098	0.099	0.005
	PLA	-0.005	0.012	0.011	-0.022	0.025	0.013
	DNN	-0.074	0.075	0.014	-0.098	0.098	0.007

Note: RMSE, root mean square error; MC-SE, Monte Carlo standard error. DML, debiased ML of proposed method; HIMA, linear structural equation models with debiased lasso and false discovery rate control (Zhang et al., 2021); Bayes, Bayesian linear mixed models with continuous shrinkage (Song et al., 2020); PLA, partially linear additive models with splines and regularization (Cai et al., 2022); DNN, partially linear models with deep neural networks and regularization (Wang and Huang, 2024).

Table S6: Mediator selection results with small coefficients under linear and nonlinear confounding structures with $n = 200$ and $p = 100$.

	linear		nonlinear	
	FPR	FNR	FPR	FNR
DML	0.009	<0.001	0.009	0.005
HIMA	0.011	<0.001	0.152	0.770
Bayes	<0.001	1.000	0.001	1.000
PLA	0.001	<0.001	0.009	<0.001
DNN	0.008	0.620	0.002	0.915

Note: FPR, false positive rate; FNR, false negative rate. DML, debiased ML of proposed method; HIMA, linear structural equation models with debiased lasso and false discovery rate control (Zhang et al., 2021); Bayes, Bayesian linear mixed models with continuous shrinkage (Song et al., 2020); PLA, partially linear additive models with splines and regularization (Cai et al., 2022); DNN, partially linear models with deep neural networks and regularization (Wang and Huang, 2024).

We have added a simulation setting that closely mirrors the real data structure to make our results more convincing. Specifically, we used the same sample size (431) and the same confounders (Z^1 to Z^{10}) as in the real data. The regression coefficients match the results from the real data, as shown in Table 4 of the main article. Additionally, we set $p = 5000$ mediators, and the nonlinear confounding structures are defined as $f(\boldsymbol{Z}_i) = g(\boldsymbol{Z}_i) = h^j(\boldsymbol{Z}_i) = \mathbb{I}(Z_i^1 > 0.5) + \mathbb{I}(Z_i^3 > 0.5)$ for $j = 1, ..., p$.

Table S7 presents the estimation results for the mediation effect, while Table S8 presents the mediator selection results. Note that some methods yield a zero MC-SE for the estimated mediation effect because, during the screening step in each simulation, the corresponding mediator is excluded. Consequently, the estimated mediation effect always remains zero. The results indicate that our proposed DML method outperforms other approaches, demonstrating that our method performs effectively in the real data setting.

Table S7: Estimation results for the mediation effect in the real data setting under linear and nonlinear confounding structures with $n = 431$ and $p = 5000$.

		Bias	RMSE	MC-SE		Bias	RMSE	MC-SE
DML	$\beta^1\gamma^1$	0.011	0.015	0.010	$\beta^6\gamma^6$	-0.012	0.024	0.021
HIMA		0.049	0.049	0		-0.069	0.069	0
Bayes		0.049	0.049	0		-0.069	0.069	0
PLA		0.049	0.049	0		-0.069	0.069	0
DNN		0.049	0.049	0		-0.069	0.069	0
DML	$\beta^2\gamma^2$	-0.002	0.030	0.030	$\beta^7\gamma^7$	0.003	0.023	0.023
HIMA		0.205	0.343	0.280		0.097	0.173	0.146
Bayes		0.181	0.315	0.262		-0.012	0.114	0.116
PLA		0.096	0.142	0.107		-0.017	0.089	0.089
DNN		0.062	0.068	0.027		-0.022	0.025	0.012
DML	$\beta^3\gamma^3$	0.011	0.026	0.024	$\beta^8\gamma^8$	-0.002	0.022	0.022
HIMA		-0.074	0.096	0.063		-0.044	0.044	0
Bayes		0.021	0.095	0.095		-0.044	0.044	0
PLA		-0.082	0.100	0.058		-0.044	0.044	0
DNN		0.060	0.061	0.005		-0.044	0.044	0
DML	$\beta^4\gamma^4$	0.010	0.024	0.022	$\beta^9\gamma^9$	0.004	0.021	0.021
HIMA		-0.070	0.089	0.055		0.080	0.150	0.129
Bayes		0.025	0.073	0.070		0.086	0.172	0.152
PLA		-0.089	0.117	0.077		0.045	0.045	0
DNN		0.053	0.054	0.006		0.050	0.053	0.018
DML	$\beta^5\gamma^5$	0.007	0.015	0.014				
HIMA		0.050	0.050	0				
Bayes		0.050	0.050	0				
PLA		0.050	0.050	0				
DNN		0.050	0.050	0				

Note: RMSE, root mean square error; MC-SE, Monte Carlo standard error. Some methods yield a zero MC-SE for the estimated mediation effect because, during the screening step in each simulation, the corresponding mediator is excluded. Consequently, the estimated mediation effect always remains zero. DML, debiased ML of proposed method; HIMA, linear structural equation models with debiased lasso and false discovery rate control (Zhang et al., 2021); Bayes, Bayesian linear mixed models with continuous shrinkage (Song et al., 2020); PLA, partially linear additive models with splines and regularization (Cai et al., 2022); DNN, partially linear models with deep neural networks and regularization (Wang and Huang, 2024).

Table S8: Mediator selection results in the real data setting under linear and nonlinear confounding structures with $n = 431$ and $p = 5000$.

	FPR	FNR	Type I	Power
DML	0.001	0.464	0.001	0.536
HIMA	<0.001	0.905	<0.001	0.095
Bayes	<0.001	1.000	<0.001	<0.001
PLA	<0.001	0.853	<0.001	0.147
DNN	<0.001	1.000	<0.001	<0.001

Note: FPR, false positive rate; FNR, false negative rate; Type I, type I error rate. DML, debiased ML of proposed method; HIMA, linear structural equation models with debiased lasso and false discovery rate control (Zhang et al., 2021); Bayes, Bayesian linear mixed models with continuous shrinkage (Song et al., 2020); PLA, partially linear additive models with splines and regularization (Cai et al., 2022); DNN, partially linear models with deep neural networks and regularization (Wang and Huang, 2024).

S2 Additional results for ADNI data analysis

Table S9 shows the results of the selected CpG sites after screening and regularization.

Table S9: Summary of the selected CpG sites after screening and regularization.

CpG sites	$\hat{\beta}^j$ (95% CI)	$\hat{\gamma}^j$ (95% CI)	$\hat{\beta}^j\hat{\gamma}^j$ (95% CI)	p^j_{ASobel}	p^j_{Bonf}
cg14128040	0.620 (0.135,1.105)	0.100 (0.041,0.159)	0.062 (0.032,0.093)	<0.001	0.002
cg03099208	-1.216 (-2.128,-0.303)	0.048 (0.017,0.079)	-0.058 (-0.087,-0.029)	<0.001	0.003
cg13885201	-0.819 (-1.481,-0.158)	0.059 (0.016,0.102)	-0.048 (-0.075,-0.022)	<0.001	0.010
cg11980435	-3.868 (-7.607,-0.130)	-0.018 (-0.028,-0.007)	0.069 (0.030,0.108)	0.001	0.017
cg27398948	3.934 (0.254,7.615)	-0.011 (-0.019,-0.003)	-0.045 (-0.071,-0.019)	0.001	0.025
cg17603321	5.096 (0.223,9.969)	-0.009 (-0.016,-0.003)	-0.048 (-0.077,-0.020)	0.001	0.026
cg27304332	1.267 (-0.052,2.585)	-0.038 (-0.060,-0.016)	-0.049 (-0.078,-0.020)	0.001	0.030
cg12712270	-5.739 (-11.350,-0.127)	-0.008 (-0.013,-0.003)	0.044 (0.018,0.071)	0.001	0.030
cg12345696	2.481 (-0.188,5.150)	-0.020 (-0.032,-0.008)	-0.050 (-0.080,-0.019)	0.001	0.043
cg09697494	-3.370 (-6.610,-0.130)	0.011 (0.001,0.020)	-0.036 (-0.059,-0.012)	0.003	0.083
cg09532113	-3.925 (-8.844,0.993)	0.010 (0.004,0.017)	-0.039 (-0.067,-0.012)	0.005	0.166
cg21582311	2.299 (-0.899,5.497)	-0.015 (-0.024,-0.006)	-0.034 (-0.060,-0.008)	0.010	0.315
cg03551796	1.905 (-0.799,4.609)	-0.018 (-0.029,-0.008)	-0.035 (-0.062,-0.008)	0.011	0.327
cg01412404	-0.423 (-0.700,-0.145)	-0.234 (-0.338,-0.130)	0.099 (0.021,0.177)	0.013	0.413
cg05639690	1.024 (0.394,1.653)	0.086 (0.042,0.131)	0.088 (0.017,0.159)	0.015	0.452
cg23829267	-2.611 (-6.702,1.480)	0.012 (0.003,0.020)	-0.030 (-0.056,-0.004)	0.023	0.723
cg00964997	1.420 (0.357,2.484)	0.058 (0.031,0.085)	0.082 (0.009,0.155)	0.027	0.830
cg05002512	5.676 (2.600,8.752)	-0.013 (-0.023,-0.004)	-0.075 (-0.141,-0.008)	0.028	0.875
cg12305251	-4.897 (-8.924,-0.871)	0.015 (0.008,0.023)	-0.076 (-0.147,-0.004)	0.037	1.000
cg03340261	4.622 (0.370,8.874)	-0.013 (-0.019,-0.006)	-0.058 (-0.120,0.004)	0.067	1.000
cg27227619	-2.469 (-5.092,0.153)	0.021 (0.010,0.032)	-0.052 (-0.113,0.009)	0.098	1.000
cg16657759	1.516 (-0.131,3.163)	-0.034 (-0.052,-0.015)	-0.051 (-0.112,0.011)	0.106	1.000
cg19222405	-5.224 (-11.241,0.794)	0.009 (0.004,0.014)	-0.048 (-0.109,0.013)	0.121	1.000
cg24567464	2.252 (-0.453,4.957)	0.025 (0.012,0.039)	0.057 (-0.018,0.132)	0.136	1.000
cg12164693	-2.041 (-4.512,0.430)	-0.022 (-0.033,-0.010)	0.044 (-0.014,0.103)	0.139	1.000
cg26536219	0.732 (-0.185,1.648)	-0.060 (-0.091,-0.029)	-0.044 (-0.103,0.016)	0.149	1.000
cg01343768	-0.813 (-1.949,0.322)	0.053 (0.027,0.080)	-0.043 (-0.107,0.021)	0.186	1.000
cg18868977	3.080 (-1.241,7.401)	0.015 (0.007,0.023)	0.047 (-0.023,0.117)	0.191	1.000
cg03070187	-0.629 (-1.633,0.374)	0.052 (0.022,0.081)	-0.033 (-0.088,0.023)	0.247	1.000
cg07847101	-0.619 (-1.941,0.703)	0.047 (0.024,0.071)	-0.029 (-0.093,0.035)	0.371	1.000
cg22283115	0.217 (-0.448,0.883)	-0.124 (-0.172,-0.075)	-0.027 (-0.110,0.056)	0.526	1.000

Figure S1 shows the relationships between the AD outcome and seven continuous confounders, while Figure S2 depicts the relationship between a selected mediator (cg13885201) and the same set of confounders. The plots indicate that these relationships may be nonlin-

ear, highlighting the importance of modeling nonlinear confounding effects.

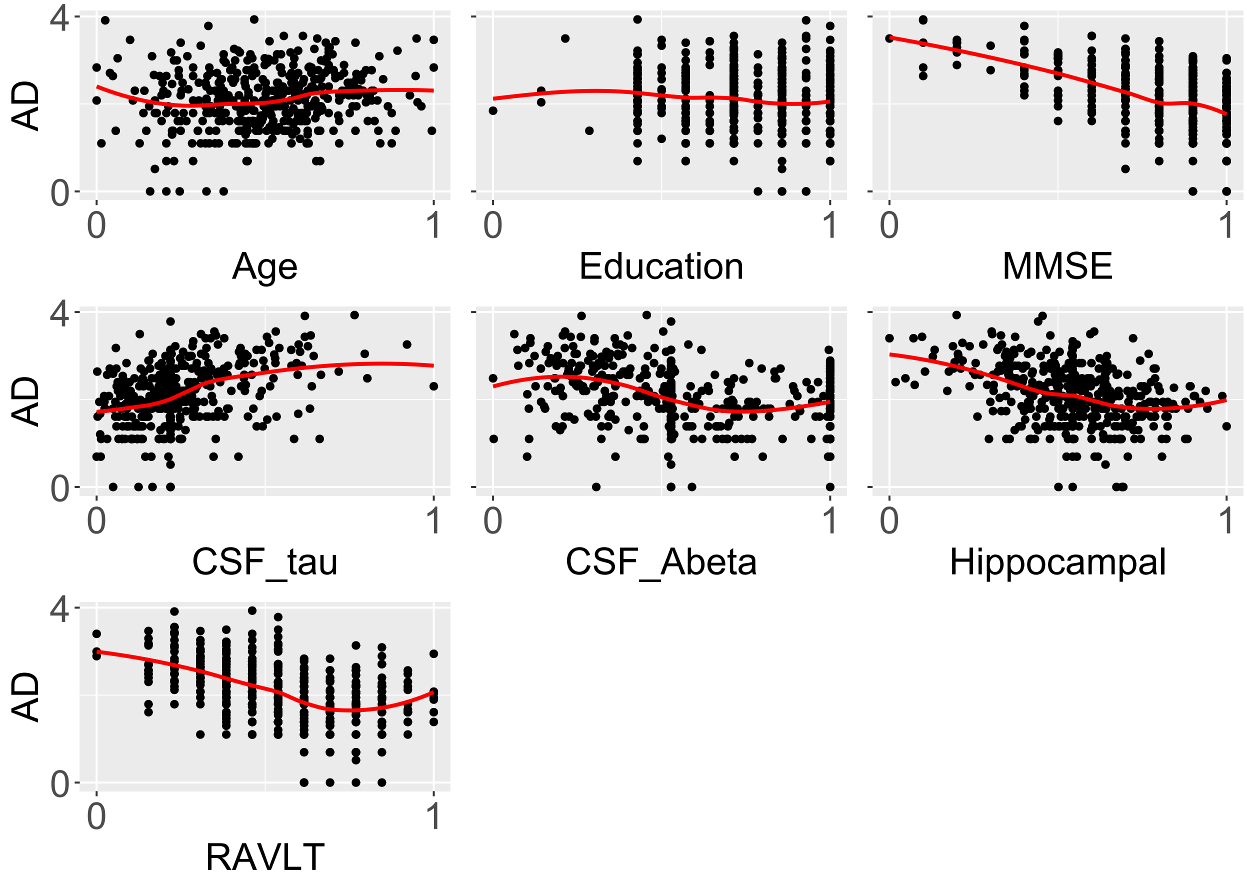

Figure S1: Scatter plots with LOESS (locally estimated scatterplot smoothing) lines to show the relationships between the AD outcome and seven continuous confounders (age, education, Mini-Mental State Examination score (MMSE), cerebrospinal fluid tau protein level (CSF_tau), cerebrospinal fluid Aβ42 level (CSF_Abeta), hippocampal volume, and Rey Auditory-Verbal Learning Test score (RAVLT)).

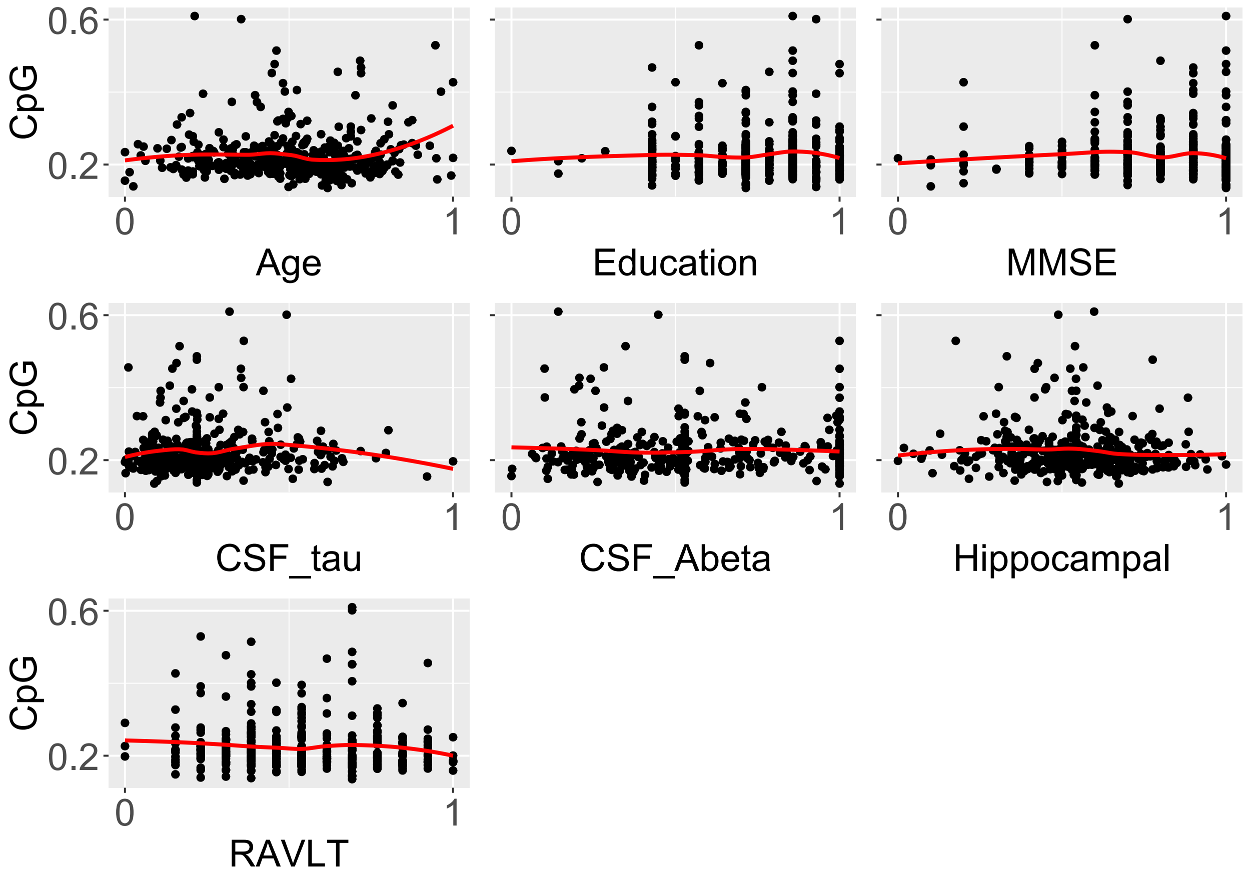

Figure S2: Scatter plots with LOESS (locally estimated scatterplot smoothing) lines to show the relationships between a selected mediator (cg13885201) and seven continuous confounders (age, education, Mini-Mental State Examination score (MMSE), cerebrospinal fluid tau protein level (CSF_tau), cerebrospinal fluid Aβ42 level (CSF_Abeta), hippocampal volume, and Rey Auditory-Verbal Learning Test score (RAVLT)).

Our estimated functions $\hat{f}(\boldsymbol{Z}_i)$ and $\hat{g}^j(\boldsymbol{Z}_i)$ lie in a q-dimensional space (with q-dimensional $\boldsymbol{Z}_i$). This differs greatly from Cai et al. (2022), where they assume the additive structures of confounding functions as $\hat{f}(\boldsymbol{Z}_i) = \sum_{k=1}^{q} \hat{f}^k\left(Z_i^k\right)$ and $\hat{g}^j(\boldsymbol{Z}_i) = \sum_{k=1}^{q} \hat{g}^{jk}\left(Z_i^k\right)$, making it relatively straightforward to visualize each dimension separately.

To better visualize our multidimensional $\hat{f}(\boldsymbol{Z}_i)$ and $\hat{g}^j(\boldsymbol{Z}_i)$ in the real data analysis, we employ partial dependence plots (Greenwell, 2017), which depict the relationship between a predictor and the response while averaging out the effects of other predictors. Figure S3 presents the partial dependence plots of $\hat{f}(\boldsymbol{Z}_i)$ with respect to seven continuous confounders, while Figure S4 shows the partial dependence plot for $\hat{g}^j(\boldsymbol{Z}_i)$ (cg13885201). The curves

indicate that the confounders exhibit complex nonlinear effects.

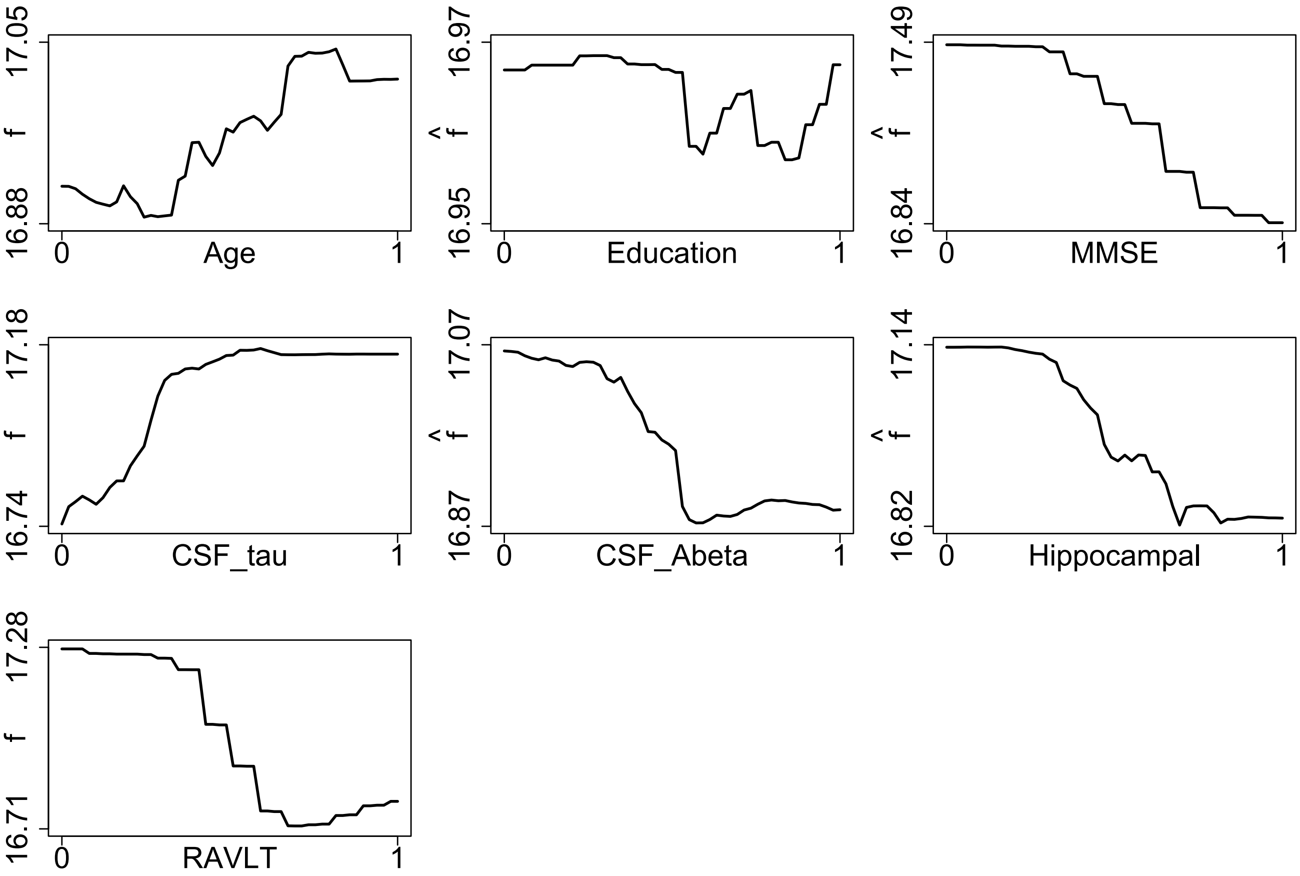

Figure S3: Partial dependence plots of $\hat{f}(\boldsymbol{Z}_i)$ with respect to seven continuous confounders (age, education, Mini-Mental State Examination score (MMSE), cerebrospinal fluid tau protein level (CSF_tau), cerebrospinal fluid Aβ42 level (CSF_Abeta), hippocampal volume, and Rey Auditory-Verbal Learning Test score (RAVLT)).

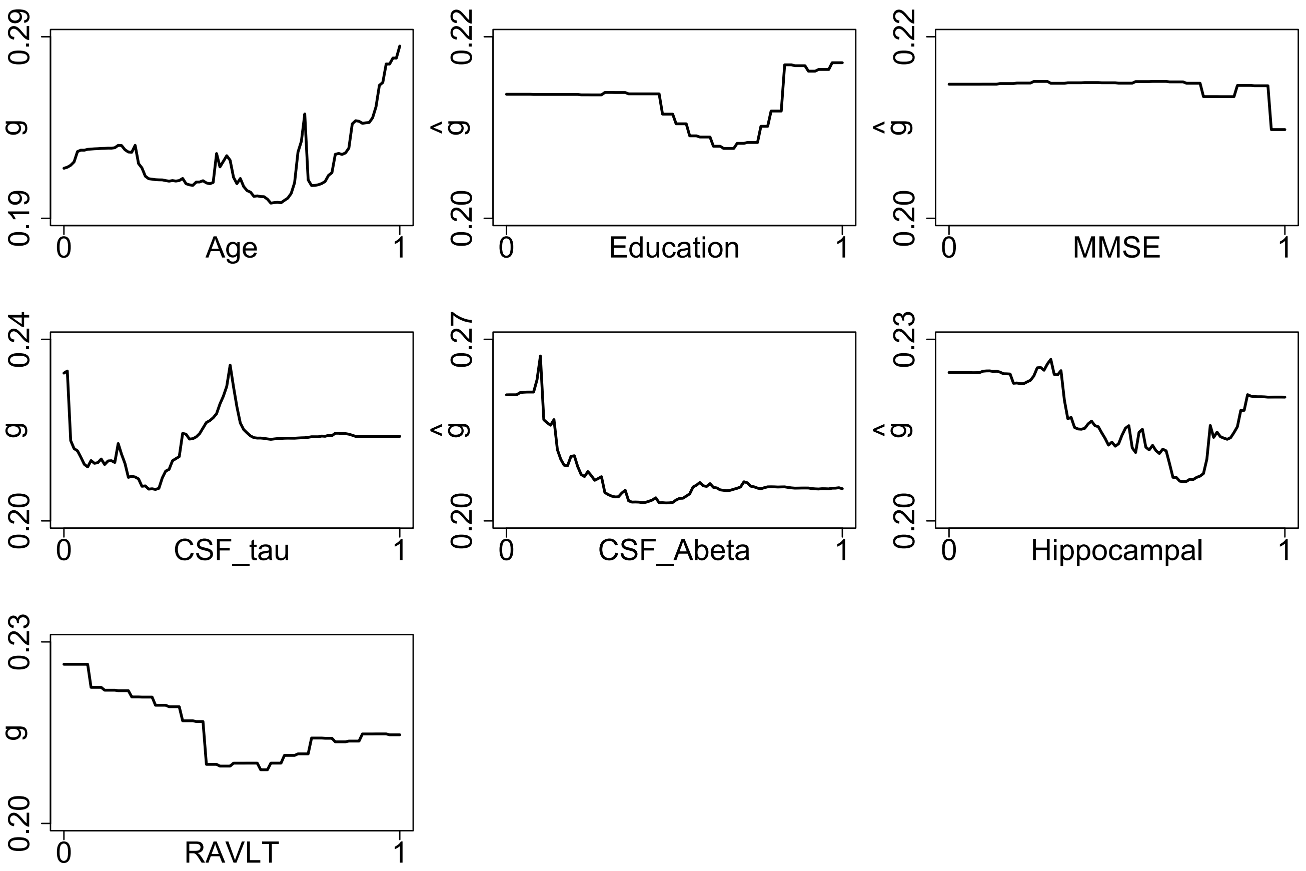

Figure S4: Partial dependence plots of $\hat{g}^j(\boldsymbol{Z}_i)$ for a selected mediator (cg13885201) with respect to seven continuous confounders (age, education, Mini-Mental State Examination score (MMSE), cerebrospinal fluid tau protein level (CSF_tau), cerebrospinal fluid Aβ42 level (CSF_Abeta), hippocampal volume, and Rey Auditory-Verbal Learning Test score (RAVLT)).

S3 A comparison between the proposed DML method and the DNN method of Wang and Huang (2024)

The goal is to estimate the regression coefficients in the structural equation models as follows:

$$Y_i = \alpha X_i + \boldsymbol{\beta}^T \boldsymbol{M}_i + f(\boldsymbol{Z}_i) + e_i, \tag{1}$$

$$M^j = \gamma^j X_i + g^j(\boldsymbol{Z}_i) + \varepsilon^j, \ (j = 1, ..., p). \tag{2}$$

To achieve this, Wang and Huang (2024) proposed incorporating deep neural networks directly into the modeling of (1) and (2) to capture the nonlinear effects of confounders, while utilizing regularization to handle the high-dimensional mediators. Their objective functions are

$$\left(\hat{\alpha}, \hat{\boldsymbol{\beta}}, \hat{f}(\cdot)\right) = \underset{\alpha\in\mathbb{R},\boldsymbol{\beta}\in\mathbb{R}^p, f(\cdot)\in\mathscr{F}}{\operatorname{argmin}} \frac{1}{n}\sum_{i=1}^{n}\left(Y_i - \alpha X_i - \boldsymbol{\beta}^T\boldsymbol{M}_i - f(\boldsymbol{Z}_i)\right)^2 + \sum_{j=1}^{p} P_\lambda\left(\left|\beta^j\right|\right), \quad (3)$$

$$\left(\hat{\gamma}^j, \hat{g}^j(\cdot)\right) = \underset{\gamma^j\in\mathbb{R}, g^j(\cdot)\in\mathscr{F}}{\operatorname{argmin}} \frac{1}{n}\sum_{i=1}^{n}\left(M^j - \gamma^j X_i - g^j(\boldsymbol{Z}_i)\right)^2,\ (j = 1, ..., p),$$

where the optimization problem in (3) involves high-dimensional inputs, including mediators and confounders, potentially increasing computational complexity. Furthermore, the regularization of $\boldsymbol{\beta}$ and the approximation of $f(\boldsymbol{Z}_i)$ are mixed together, which may lead to computational instability.

In contrast, for our method, we first apply a conditional expectation to both sides of (1) to obtain

$$\mathbb{E}(Y_i|\boldsymbol{Z}_i) = \alpha\mathbb{E}(X_i|\boldsymbol{Z}_i) + \boldsymbol{\beta}^T\mathbb{E}(\boldsymbol{M}_i|\boldsymbol{Z}_i) + f(\boldsymbol{Z}_i). \quad (4)$$

By subtracting (4) from (1), we have

$$Y_i - \ell(\boldsymbol{Z}_i) = \alpha(X_i - \hbar(\boldsymbol{Z}_i)) + \boldsymbol{\beta}^T(\boldsymbol{M}_i - \boldsymbol{g}(\boldsymbol{Z}_i)) + e_i, \quad (5)$$

where $\ell(\boldsymbol{Z}_i) = \mathbb{E}(Y_i|\boldsymbol{Z}_i)$, $\boldsymbol{g}(\boldsymbol{Z}_i) = (g^1(\boldsymbol{Z}_i), ..., g^p(\boldsymbol{Z}_i))^T$ with $g^j(\boldsymbol{Z}_i) = \mathbb{E}\left(M_i^j|\boldsymbol{Z}_i\right)$ for $j = 1, ..., p$, and $\hbar(\boldsymbol{Z}_i) = \mathbb{E}(X_i|\boldsymbol{Z}_i)$ are nuisance functions. We apply ML approach to the k-th fold dataset $\{\boldsymbol{D}_i\}_{i\notin\mathcal{I}_k}$ to obtain estimates of the nuisance functions $\left(\hat{\ell}_k(\cdot), \hat{\boldsymbol{g}}_k(\cdot)^T, \hat{\hbar}_k(\cdot)\right)^T$. In this step, any ML approach can be applied, with the input involving only the confounders $\boldsymbol{Z}_i$ and not the mediators $\boldsymbol{M}_i$. This reduces complexity and enhances both flexibility and stability. Then, the coefficients α and $\boldsymbol{\beta}$ in (5) can be estimated using the regularized least squares with sample splitting:

$$\left(\hat{\alpha}, \hat{\boldsymbol{\beta}}\right) = \underset{\alpha\in\mathbb{R},\boldsymbol{\beta}\in\mathbb{R}^p}{\operatorname{argmin}} \frac{1}{n}\sum_{k=1}^{K}\sum_{i\in\mathcal{I}_k}\left(Y_i - \hat{\ell}_k(\boldsymbol{Z}_i) - \alpha\left(X_i - \hat{\hbar}_k(\boldsymbol{Z}_i)\right) - \boldsymbol{\beta}^T\left(\boldsymbol{M}_i - \hat{\boldsymbol{g}}_k(\boldsymbol{Z}_i)\right)\right)^2 + \sum_{j=1}^{p} P_\lambda\left(\left|\beta^j\right|\right), \quad (6)$$

Similarly, for the coefficients $\boldsymbol{\gamma}$ in (2):

$$\hat{\gamma}^j = \underset{\gamma^j\in\mathbb{R}}{\operatorname{argmin}} \frac{1}{n}\sum_{k=1}^{K}\sum_{i\in\mathcal{I}_k}\left(M^j - \hat{g}_k^j(\boldsymbol{Z}_i) - \gamma^j\left(X_i - \hat{\hbar}_k(\boldsymbol{Z}_i)\right)\right)^2,\ (j = 1, ..., p). \quad (7)$$

Both (6) and (7) can be easily implemented, for example, using the glmnet package in R. In summary, the proposed method separates the steps for regularizing $\boldsymbol{\beta}$ and approximating complex confounding functions, requiring fewer inputs when applying ML approaches. This separation may reduce computational complexity and improve stability.

Table S10 presents the average running time per replication and the average bias of all estimated mediation effects. The proposed DML method achieves both a shorter running time and smaller bias compared to the DNN method proposed by Wang and Huang (2024).

Table S10: Averaged simulation time (seconds per replication) and bias of all estimated mediation effects under linear and nonlinear confounding structures.

	linear				nonlinear			
	$n = 200$ $p = 100$		$n = 200$ $p = 600$		$n = 200$ $p = 100$		$n = 200$ $p = 600$	
	Time	Bias	Time	Bias	Time	Bias	Time	Bias
DML	64.781	-0.013	290.947	-0.022	65.182	-0.063	298.827	-0.059
HIMA	27.359	0.219	28.100	0.222	29.859	-0.797	29.705	-0.820
Bayes	13.266	0.003	13.855	0.000	13.207	-0.962	13.829	-0.965
PLA	45.942	-0.031	45.928	-0.100	48.458	-0.260	42.378	-0.237
DNN	561.550	-0.628	545.408	-0.636	559.414	-0.962	554.426	-0.965
	$n = 500$ $p = 100$		$n = 500$ $p = 600$		$n = 500$ $p = 100$		$n = 500$ $p = 600$	
	Time	Bias	Time	Bias	Time	Bias	Time	Bias
DML	177.404	0.017	896.258	0.016	179.915	-0.008	922.355	-0.008
HIMA	30.635	0.206	81.663	0.202	31.881	-0.418	74.644	-0.929
Bayes	59.084	0.001	58.599	-0.007	58.768	-0.977	58.920	-0.987
PLA	54.723	-0.004	56.134	-0.048	70.083	-0.271	51.878	-0.258
DNN	2222.034	-0.623	2140.809	-0.622	2264.658	-0.970	2249.692	-0.982

Note: bias is averaged across $\beta^1\gamma^1$ to $\beta^4\gamma^4$. DML, debiased ML of proposed method; HIMA, linear structural equation models with debiased lasso and false discovery rate control (Zhang et al., 2021); Bayes, Bayesian linear mixed models with continuous shrinkage (Song et al., 2020); PLA, partially linear additive models with splines and regularization (Cai et al., 2022); DNN, partially linear models with deep neural networks and regularization (Wang and Huang, 2024).

S4 A comparison between the proposed DML method and the DeepMed method of Xu et al. (2022)

The DeepMed method proposed by Xu et al. (2022) is primarily designed for mediation analysis with a binary exposure and a single mediator. Specifically, let $X_i \in \{0, 1\}$, $Y_i \in \mathbb{R}$, $M_i \in \mathbb{R}$, and $\boldsymbol{Z}_i \in \mathbb{R}^q$ represent the binary exposure, outcome, mediator, and q-dimensional confounders for the i-th subject $(i = 1, ..., n)$. Let $Y^{x,m}$ be the potential outcome that would have been observed if X was set to x and M was set to m, and M^x be the potential mediator that would have been observed if X was set to x. The natural direct effect for $x = 1$ versus $x = 0$ is $\mathbb{E}\left(Y^{1,m^0} - Y^{0,m^0}\right)$, and the natural indirect effect is $\mathbb{E}\left(Y^{1,m^1} - Y^{1,m^0}\right)$.

Estimating natural direct effect and indirect effect can be reduced to estimating $\psi(x, x') = \mathbb{E}\left(Y^{x,m^{x'}}\right)$ for $x, x' \in \{0, 1\}$. Xu et al. (2022) construct cross-fitted forms of the empirical moments to estimate $\psi(x, x')$. Specifically, subjects $\{1, ..., n\}$ are randomly divided into K folds $\{\mathcal{I}_k\}_{k=1}^K$ and the sizes for folds 1 to K are $(\lfloor n/K \rfloor, ..., \lfloor n/K \rfloor, n - (K-1)\lfloor n/K \rfloor)$. The DeepMed estimator $\hat{\psi}(x, x')$ can be obtained by solving the following empirical moments:

$$\hat{\psi}(x, x') = \frac{1}{n}\sum_{k=1}^{K}\sum_{i\in\mathcal{I}_k}\varphi_{x,x'}\left(\boldsymbol{D}_i; \hat{\mathscr{f}}_k(\cdot), \hat{\mathscr{g}}_k(\cdot), \hat{\mathscr{h}}_k(\cdot)\right),$$

$$\varphi_{x,x'}(\boldsymbol{D}_i; \mathscr{f}(\cdot), \mathscr{g}(\cdot), \mathscr{h}(\cdot)) = \frac{\mathbb{I}(X_i = x)\, \mathscr{g}(M_i, \boldsymbol{Z}_i, x')}{\mathscr{h}(x, \boldsymbol{Z}_i)\, \mathscr{g}(M_i, \boldsymbol{Z}_i, x)}(Y_i - \mathscr{f}(\boldsymbol{Z}_i, x, M_i))$$
$$+\left(1 - \frac{\mathbb{I}(X_i = x')}{\mathscr{h}(x', \boldsymbol{Z}_i)}\right)\int \mathscr{f}(\boldsymbol{Z}_i, x, M)\, \mathscr{g}(M, \boldsymbol{Z}_i, x')\, dM + \frac{\mathbb{I}(X_i = x')}{h(x', \boldsymbol{Z}_i)}\mathscr{f}(\boldsymbol{Z}_i, x, M_i),$$

where $\mathscr{h}(x, \boldsymbol{Z}_i) = \mathbb{D}(x|\boldsymbol{Z}_i)$ is the conditional density function of $X = x$ given $\boldsymbol{Z}_i$, $\mathscr{g}(M_i, \boldsymbol{Z}_i, x) = \mathbb{D}(M_i|\boldsymbol{Z}_i, x)$ is the conditional density function of M_i given $\boldsymbol{Z}_i$ and $X = x$, and $\mathscr{f}(\boldsymbol{Z}_i, x, M_i) = \mathbb{E}(Y_i|\boldsymbol{Z}_i, x, M_i)$ is the conditional expectation of M_i given $\boldsymbol{Z}_i$, $X = x$, and M_i, all of which are nuisance functions, and $\hat{\mathscr{h}}_k(\cdot)$, $\hat{\mathscr{g}}_k(\cdot)$, and $\hat{\mathscr{f}}_k(\cdot)$ are ML estimators based solely on the subset of data $\{\boldsymbol{D}_i\}_{i\notin\mathcal{I}_k}$.

For the proposed debiased ML (DML) framework, we approach it from the perspective of structural equation modeling:

$$Y_i = \alpha X_i + \beta M_i + f(\boldsymbol{Z}_i) + e_i,$$

$$M_i = \gamma X_i + g(\boldsymbol{Z}_i) + \varepsilon_i,$$

where, under the assumptions of sequential ignorability and other conditions (Imai et al., 2010), regression coefficients can be causally interpreted, that the natural direct effect

for $x = 1$ versus $x = 0$ is $\mathbb{E}\left(Y^{1,m^0} - Y^{0,m^0}\right) = \alpha$, and the natural indirect effect is $\mathbb{E}\left(Y^{1,m^1} - Y^{1,m^0}\right) = \beta\gamma$.

Estimating natural direct effect and indirect effect can be reduced to estimating α, β, and γ. The sample splitting step is the same as that in DeepMed, while the DML estimator $\left(\hat{\alpha}, \hat{\beta}, \hat{\gamma}\right)$ can be obtained by solving the following empirical moments:

$$\frac{1}{n}\sum_{k=1}^{K}\sum_{i\in\mathcal{I}_k}\varphi\left(\boldsymbol{D}_i;\alpha,\beta,\hat{\ell}_k(\cdot),\hat{g}_k(\cdot),\hat{h}_k(\cdot)\right) = \boldsymbol{0}, \tag{8}$$

$$\frac{1}{n}\sum_{k=1}^{K}\sum_{i\in\mathcal{I}_k}\phi\left(\boldsymbol{D}_i;\gamma,\hat{g}_k(\cdot),\hat{h}_k(\cdot)\right) = 0, \tag{9}$$

$$\varphi\left(\boldsymbol{D}_i;\alpha,\beta,\ell(\cdot),g(\cdot),h(\cdot)\right) = \left\{Y_i - \ell(\boldsymbol{Z}_i) - \alpha\left(X_i - h(\boldsymbol{Z}_i)\right) - \beta\left(M_i - g(\boldsymbol{Z}_i)\right)\right\}\begin{pmatrix} X_i - h(\boldsymbol{Z}_i) \\ M_i - g(\boldsymbol{Z}_i)\end{pmatrix},$$

$$\phi\left(\boldsymbol{D}_i;\gamma,g(\cdot),h(\cdot)\right) = \left\{M_i - g(\boldsymbol{Z}_i) - \gamma\left(X_i - h(\boldsymbol{Z}_i)\right)\right\}\left(X_i - h(\boldsymbol{Z}_i)\right),$$

where $h(\boldsymbol{Z}_i) = \mathbb{E}(X_i|\boldsymbol{Z}_i)$, $g(\boldsymbol{Z}_i) = \mathbb{E}(M_i|\boldsymbol{Z}_i)$, and $\ell(\boldsymbol{Z}_i) = \mathbb{E}(Y_i|\boldsymbol{Z}_i)$ are nuisance functions, and $\hat{h}_k(\cdot)$, $\hat{g}_k(\cdot)$, and $\hat{\ell}_k(\cdot)$ are ML estimators based solely on the subset of data $\{\boldsymbol{D}_i\}_{i\notin\mathcal{I}_k}$.

In summary, both the DeepMed and DML methods utilize the concepts of orthogonality score functions and sample splitting. The DeepMed method derives the orthogonality score function based on the efficient influence function for $\psi(x, x')$ (Tchetgen Tchetgen and Shpitser, 2012), while the DML method obtains the orthogonality score function based on the regression model and the double residual semiparametric regression estimator for α, β, and γ (Robinson, 1988). Both methods are grounded in the semi-parametric literature, but in comparison to DeepMed, the key differences and advantages of our DML method are as follows:

(1) The DeepMed method is limited to binary exposure variables, whereas our DML method allows for a broader range of exposure types, including both discrete and continuous variables.

(2) The nuisance functions in DeepMed are $h(x, \boldsymbol{Z}_i) = \mathbb{D}(x|\boldsymbol{Z}_i)$, $g(M_i, \boldsymbol{Z}_i, x) = \mathbb{D}(M_i|\boldsymbol{Z}_i, x)$, and $\ell(\boldsymbol{Z}_i, x, M_i) = \mathbb{E}(Y_i|\boldsymbol{Z}_i, x, M_i)$, which differ from the nuisance functions $h(\boldsymbol{Z}_i) = \mathbb{E}(X_i|\boldsymbol{Z}_i)$, $g(\boldsymbol{Z}_i) = \mathbb{E}(M_i|\boldsymbol{Z}_i)$, and $\ell(\boldsymbol{Z}_i) = \mathbb{E}(Y_i|\boldsymbol{Z}_i)$ in DML. When using ML to estimate $\ell(\boldsymbol{Z}_i, x, M_i) = \mathbb{E}(Y_i|\boldsymbol{Z}_i, x, M_i)$ in DeepMed, the predictors include the exposure, mediator, and confounders. This may not pose a great challenge when the number of mediators is small; however, as the number of mediators increases, obtaining an accurate estimator

for $\mathbb{E}(Y_i|\boldsymbol{Z}_i, x, M_i)$ becomes increasingly difficult. In contrast, the corresponding nuisance function in DML is $\ell(\boldsymbol{Z}_i) = \mathbb{E}(Y_i|\boldsymbol{Z}_i)$, where the predictors only include the confounders, even in the high-dimensional mediator setting, making it easier to obtain a more accurate estimator for $\mathbb{E}(Y_i|\boldsymbol{Z}_i)$.

(3) The DeepMed method is primarily designed for mediation analysis with a single mediator. However, defining the causal parameter $\psi(x, x') = \mathbb{E}\left(Y^{x,m^{x'}}\right)$ in the high-dimensional mediator setting and selecting important mediators remain open questions. In contrast, our DML framework offers a comprehensive approach for high-dimensional mediator settings by incorporating screening and regularization techniques alongside empirical moments (8) and (9) to accurately select mediators. Additionally, it provides statistical inference tools for assessing the contributions of these mediators.

References

Cai X, Zhu Y, Huang Y et al. High-dimensional causal mediation analysis based on partial linear structural equation models. *Comput Stat Data Anal* 2022;174:107501.

Greenwell, BM. pdp: An R package for constructing partial dependence plots. *RJ* 2017;9:421–436.

Imai K, Keele L, Yamamoto T. Identification, inference and sensitivity analysis for causal mediation effects. *Stat Sci* 2010;25:51–71.

Robinson PM. Root-N-consistent semiparametric regression. *Econometrica* 1988;56:931–954.

Song Y, Zhou X, Zhang M et al. Bayesian shrinkage estimation of high dimensional causal mediation effects in omics studies. *Biometrics* 2020;76:700–710.

Tchetgen Tchetgen EJ, Shpitser I. Semiparametric theory for causal mediation analysis: Efficiency bounds, multiple robustness, and sensitivity analysis. *Ann Stat* 2012;40:1816–1845.

Wang S, Huang Y. DP2LM: Leveraging deep learning approach for estimation and hypothesis testing on mediation effects with ultra-high dimensional mediators and complex confounders. *Biostatistics* 2024;kxad037.

Xu S, Liu L, Liu Z. DeepMed: Semiparametric causal mediation analysis with debiased deep learning. *Adv Neural Inf Process Syst* 2022;25:277–300.

Zhang H, Zheng Y, Hou L et al. Mediation analysis for survival data with ultra-high dimensional mediators. *Bioinformatics* 2021;37:3815–3821.
